# Supplementary material for: Tuning hydrogenation chemistry of Pd-based heterogeneous catalysts by introducing homogeneous-like ligands
Source: Nat Commun. 2023 Jul 4;14:3944. doi: 10.1038/s41467-023-39478-2 (PMC10319742; doi:10.1038/s41467-023-39478-2)
Supplement: Supplementary file 1 — Supplementary Information [file 41467_2023_39478_MOESM1_ESM.pdf]

Supplementary Information

Tuning Hydrogenation Chemistry of Pd-based Heterogeneous Catalysts by Introducing Homogeneous-like Ligands

Jianghao Zhang,[1, 2, ‡] Wenda Hu,[1, 3, ‡] Binbin Qian,[4,5] Houqian Li,[1] Berlin Sudduth,[1] Mark Engelhard,[3] Lian Zhang,[5] Jianzhi Hu,[1,3] Junming Sun,[1]* Changbin Zhang,[2]* Hong He,[2] Yong Wang[1,3]*

[1] The Gene & Linda Voiland School of Chemical Engineering and Bioengineering, Washington State University, Pullman, WA 99164, United States

[2] State Key Joint Laboratory of Environment Simulation and Pollution Control, Research Center for Eco-environmental Sciences, Chinese Academy of Sciences, Beijing, 100085, China

[3] Institute for Integrated Catalysis, Pacific Northwest National Laboratory, Richland, WA 99352, United States

[4] School of Chemistry and Environmental Engineering, Yancheng Teachers University, Yancheng 224002, China

[5] Department of Chemical Engineering, Monash University, Clayton, Victoria 3800, Australia

[‡] These authors contributed equally: Jianghao Zhang, Wenda Hu.

**Corresponding author:*

Junming Sun: +1 509-335-1880 (phone) junming.sun@wsu.edu

Changbin Zhang: +86 010-62849194 (phone) cbzhang@rcees.ac.cn

Yong Wang: +1 509-371-6273 (phone), yong.wang@pnnl.gov

1. Supplementary Method

1.1 Synthesis of catalysts

1.1.1 Preparation of Pd/CNT

The Pd/CNT was prepared with impregnation method. $Pd(NH_3)_4(NO_3)_2$ solution (Sigma-Aldrich, 10 wt.% solution with 99.99% metal based purity) was added dropwise to carbon nanotube reaching to 5 wt.% loading of Pd. After stirring the support/solution mixture and evaporation of water to reach an even dispersion of solution over the carbon surface. The paste was dried at 80 °C overnight. The carbon ligand deposition was performed with the same procedure as that of 5Pd-C/Fe-C.

1.1.2 Depositing different amounts of carbon ligand on 5Pd/Fe

Different amounts of carbon ligand were deposited on 5Pd/Fe using the same procedure as that of 5Pd-C/Fe-C except for using different CO concentrations as 1.0 vol.% and 4.0 vol.% in H_2.

1.2 Characterization

XRD was performed on a Rigaku Miniflex II X–ray diffractometer with $Cu_{\kappa\alpha}$ radiation (λ =1.54178 Å) operating at 40 kV and 50 mA. The patterns were collected at a scanning rate of 1°/min with step-size of 0.01°.

CO pulse chemisorption experiments were conducted on Chemisorption Analyzer (Micromeritics AutoChem 2920) equipped with a quadrupole mass spectrometer (QMS, Omnistar gas analyzer GSD 301). In a typical test, 0.05 g sample was pretreated in 10% H_2/Ar for 1 h at 250 °C to simulate the catalysts during hydrotreating reaction. After cooling down to 30 °C and purging with 5%Ar/He for 30 min, 0.5 mL 10%CO/He was injected to sample repeating 15 times. The QMS signal of CO was recorded at m/z=28 and normalized by Ar (m/z=40). The dispersion was determined by assuming a chemisorption stoichiometry of one CO molecule per surface Pd atom[1]. The mean size of Pd nanoparticle (d_m) was estimated with an assumption of hemispherical morphology using the reported equation[2]:

$$d_m\ (nm) = 10^{21} \times (6 \times M \times \rho_{surface\ site}) / (D \times \rho_{Pd} \times N) \quad (1)$$

where M is the atomic weight of Pd (106.4 g/mol), $\rho_{surface\ site}$ is the surface site density of Pd (12.7 atoms/nm^2)[3], D is dispersion, ρ_{Pd} is the metal density (12.0 g/cm^3) and N as the Avogadro constant, giving d_m (nm) = 1.12/D.

Temperature programmed surface reaction with H_2 (H_2-TPSR) of Pd/C was performed using the same facility. Specifically, 0.05 g sample was loaded into a quartz tube and pretreated *in-situ* with the same procedure in the synthesis of 5Pd/Fe or 5Pd-C/Fe-C. After switching to 10 vol.% H_2/Ar and purging for 30min, the temperature was ramped to 500 °C (10 °C/min).

High resolution transmission electron microscopy (HRTEM) of the catalysts was performed using a JEM-2100F operated at 200 kV equipped with a 4k charge coupled device (CCD) camera. Transmission electron microscopy (TEM) analysis of the catalysts was performed using a FEI Technai G2 20 Twin operated at 200 kV and equipped with a 4k Eagle CCD camera. To prepare the samples for the test, several milligrams of sample was suspended into ethanol with a sonicator, then a drop of the nanoparticle suspension was dispensed onto a 3-mm copper grid with lacey carbon films. Excess ethanol was removed by an absorbent paper, and the sample was dried at room temperature for 6 hours. The mean particle diameter was calculated as previously reported[4]:

$$d_m = \sqrt{(\sum n_i \times d_i^2) / \sum n_i} \quad (2)$$

Raman spectroscopy analysis was conducted on a Horiba LabRAM HR Raman/FTIR microscope equipped with a 532 nm (Ventus LP 532) laser source and Synapse Charge Coupled Device detector (CCD). After calibration using the silica reference, the spectrum of the sample was then taken at room temperature.

X-ray absorption spectra of Pd K edge and Fe K edge were recorded at Beamline BL16A1 of NSRRC (National Synchrotron Research Radiation Centre) in Taiwan. The X-ray absorption near-edge spectroscopy (XANES) and extended X-ray absorption fine structure spectroscopy (EXAFS) data were collected in standard transmission mode at room temperature. Data was processed using ATHENA software (a part of DEMETER package).

1.3 Determining the amounts of active sites

The amounts of exposed Pd and Fe were determined by CO pulse chemisorption at ambient temperature and N_2 physisorption measuring BET surface area. A chemisorption stoichiometry of one CO molecule per surface Pd atom is assumed[1]. After determining the amounts of exposed Pd, the surface areas of Pd were calculated by 7.895×10^{-2} nm^2/Pd obtained from arithmetic average of the number densities of low index facets: (111), (100) and (110)[3]. By subtracting the area of Pd from the BET surface area of catalyst, the areas of exposed Fe were obtained, which can derive the amounts of exposed Fe based on its arithmetic average of atomic areas (7.014×10^{-2} nm^2/Fe) on (100) and (110) facets, which are dominant in Fe[5]. Since Fe is the site for C-O bond cleavage and Pd for direct hydrogenation, the turnover frequencies (TOFs) of the two reaction pathways were calculated based on Fe and Pd, respectively. For Fe, 0.1Pd/Fe and 1Pd/Fe, the TOFs were derived after a 2-h reaction. While for 5Pd/Fe, 5Pd/Fe-C and 5Pd-C/Fe-C, the products after 1-h reaction were taken to control the conversion of reactant (i.e., diphenyl ether) below 20%.

1.4 Computational details

The NMR chemical shifts were computed for the optimized structures by applying the GIAO model[6] using B3LYP/Lanl2DZ computational level[7,8]. Tetramethylsilane was used as the computational standard to correlate the calculated nuclear shielding from DFT to the observed NMR chemical shifts. The calculated shielding (σ) relates to the chemical shift (δ) by formula:

$$\delta(^{13}C) = \sigma_{calc,\,ref} - \sigma_{calc}.^{9} \quad (3)$$

The adsorption energy (E_{ads}) of DPE on Pd is defined in terms of the stabilization energy for adsorbate/substrate interaction, calculated according to the expression:

$$E_{ads} = E_{(substrate + adsorbate)} – (E_{substrate} + E_{adsorbate}) \quad (4)$$

where $E_{(substrate + adsorbate)}$ is the total energy of the optimized system for adsorbate on substrate. $E_{substrate}$ and $E_{adsorbate}$ are the energies of isolated substrate and adsorbate, respectively. The substrate in the expression is Pd cluster or the Pd-metallacycle composite. The adsorbate is DPE or metallacycle.

2. Supplementary Results

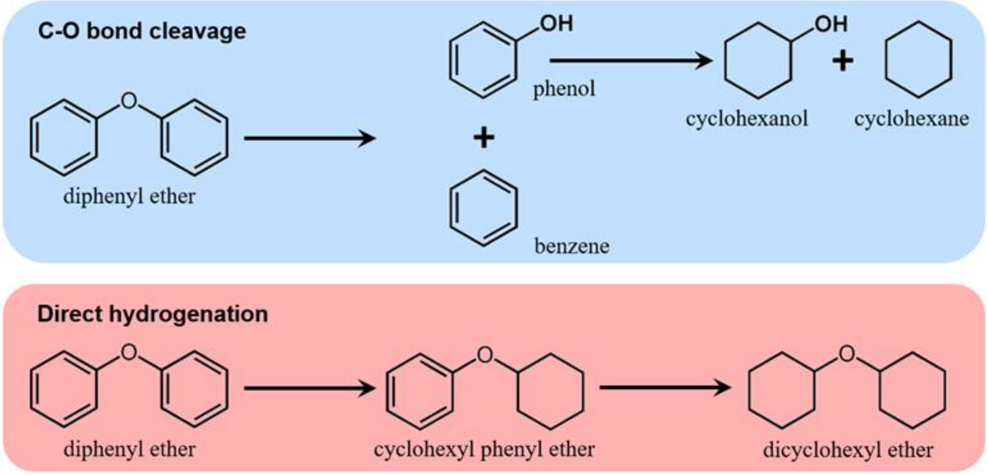

Supplementary Fig. 1 Different reaction pathways in the conversion of diphenyl ether.

Supplementary Note 1 Reaction pathways for the hydrotreating of diphenyl ether under the testing condition in this work.

Diphenyl ether (DPE), mimicking the 4-O-5 linkage in lignin, was used as modelling compound to study the Pd-Fe catalysts in deconstruction of the network of lignin. As shown in Supplementary Fig. S1, the conversion of diphenyl ether under the testing condition in this work is via two parallel reaction pathways, i.e. C-O bond cleavage forming monomers such as benzene and phenol which may be further converted to deoxygenated hydrocarbons, and direct hydrogenation producing dimers that are ring-hydrogenated without C-O bond cleavage[10,11]. In the hydrotreating of real lignin, C-O bond cleavage contributes to depolymerization of lignin, while direct hydrogenation saturates the aromatic ring with the consumption of excessive hydrogen without breaking C-O bond. Moreover, further C-O bond cleavage of the fully or partially hydrogenated polymer does not readily occur[12]. Therefore, it is desirable to selectively cleave the C-O bond in the hydrotreating process.

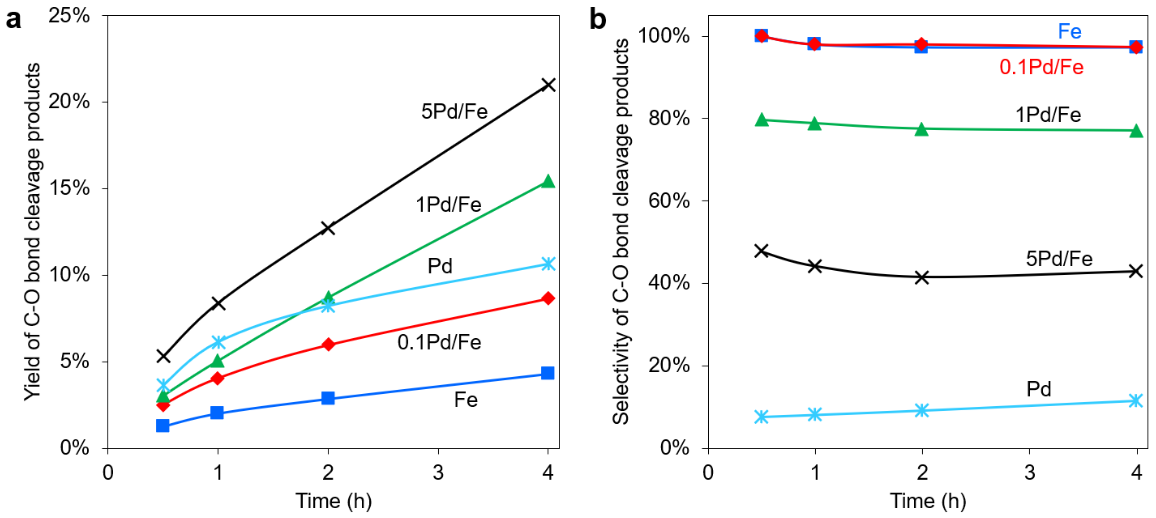

Supplementary Fig. 2 Performance of Fe, Pd and Pd-Fe catalysts in time-on-stream test. a, Yield of products from C-O bond cleavage. b, the selectivity of C-O bond cleavage products (balances are direct hydrogenation products: cyclohexyl phenyl ether and dicyclohexyl ether). Reaction condition: 0.03 g catalyst, 1.08 g DPE as reactant, 250 °C, 5.6 MPa H_2.

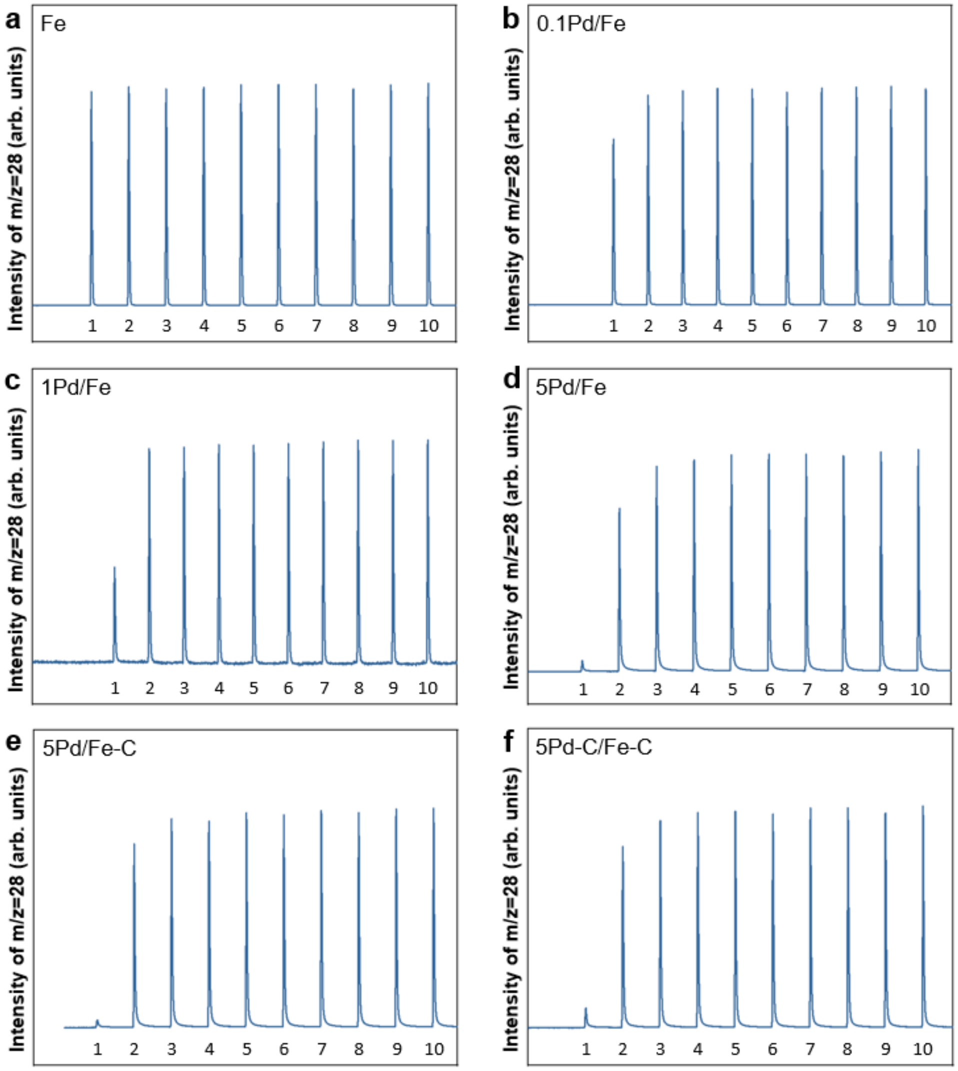

Supplementary Fig. 3 Results of CO pulse experiments at 30 °C on monometallic Fe (a), 0.1Pd/Fe (b), 1Pd/Fe (c), 5Pd/Fe (d), 5Pd/Fe-C (e) and 5Pd-C/Fe-C (f). Prior to pulsing CO, the catalysts were pretreated with $10H_2$/Ar at 250 °C for 1h to simulate the catalysts during the reaction. The signal of CO was recorded at m/z=28.

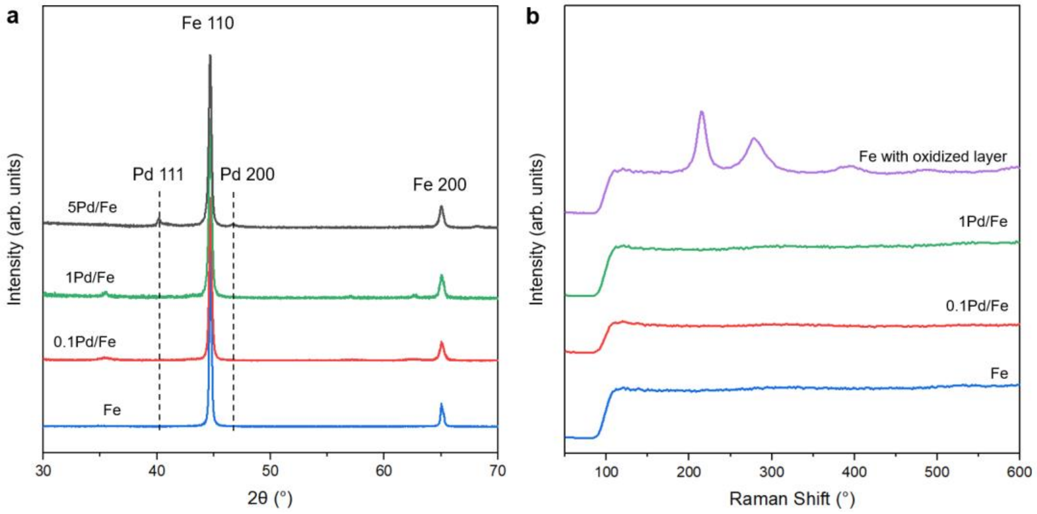

Supplementary Fig. 4 XRD patterns (a) and Raman spectra (b) of catalysts containing different amounts of Pd. The Raman spectra of Fe, 0.1Pd/Fe and 1Pd/Fe were taken after an in-situ pretreatment using 10% H_2/N_2 for 0.5 h.

The small peaks at 35.4, 56.9 and 62.5° are attributed to the diffraction of Fe_3O_4 that may be located inside the core of catalyst particle. It should be noted that the all the catalysts were reduced under same conditions except that Fe and 0.1Pd/Fe were reduced at longer time. Therefore, the core of 1Pd/Fe may not be fully reduced. In addition, compared with the oxide reference (i.e., the unpretreated Fe with oxide overlayer), no oxide peak was observed in the Raman spectra of Fe, 0.1Pd/Fe and 1Pd/Fe. This indicates that the Fe species on surface should still be in metallic state in the hydrotreating reaction, which is consistent with the in-situ XPS results in our previous study[13].

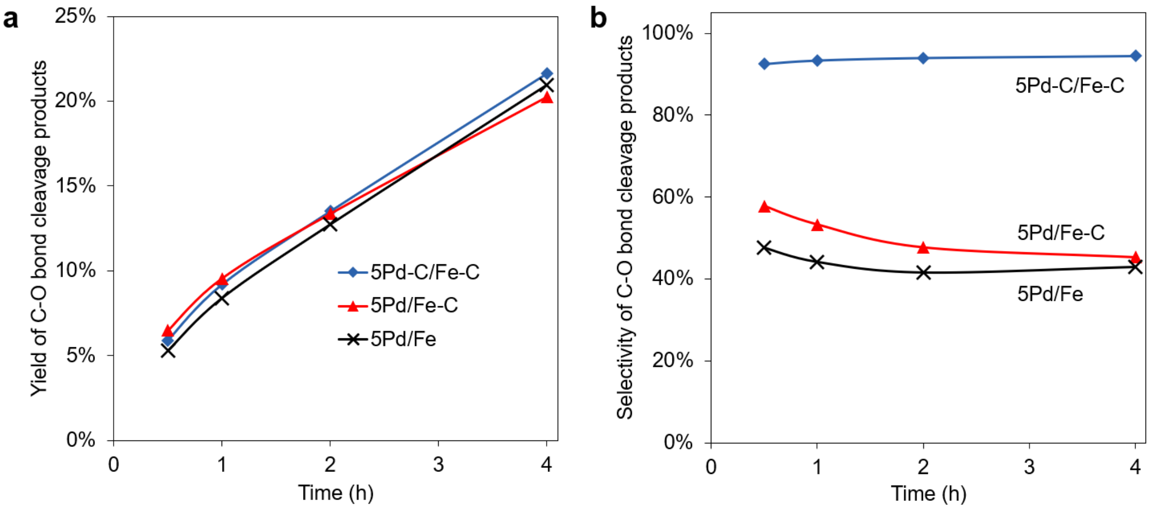

Supplementary Fig. 5 Performance of 5Pd/Fe, 5Pd/Fe-C, 5Pd-C/Fe-C in time-on-stream test. a, Yield of products from C-O bond cleavage. b, the selectivity of C-O bond cleavage products. Reaction condition: 0.03 g catalyst, 1.08 g DPE as reactant, 250 °C, 5.6 MPa H_2.

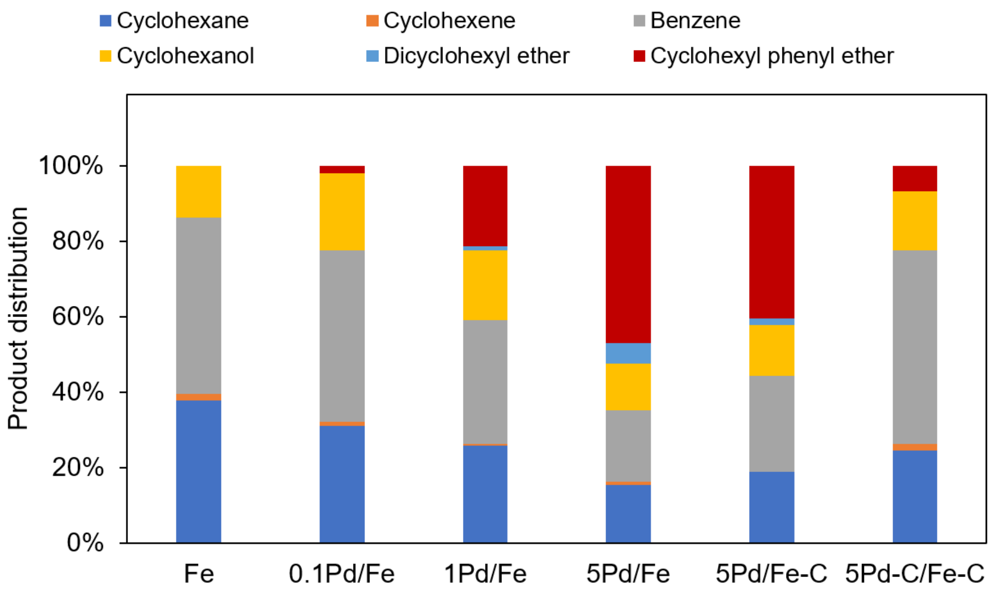

Supplementary Fig. 6 Selectivity of different products at ~10% conversion (except that the selectivity for monometallic Fe was derived at ~5% conversion).

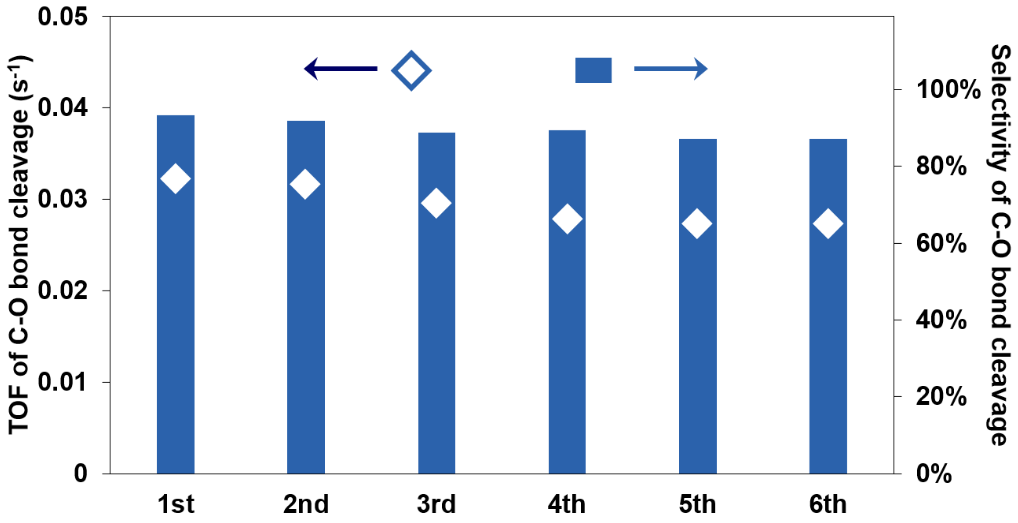

Supplementary Fig. 7 Stability test of 5Pd-C/Fe-C. Reaction condition: 0.03 g catalyst, 1h, 50 mL $C_{16}H_{34}$, 1.08 g DPE, 250 °C, 5.6 MPa H_2.

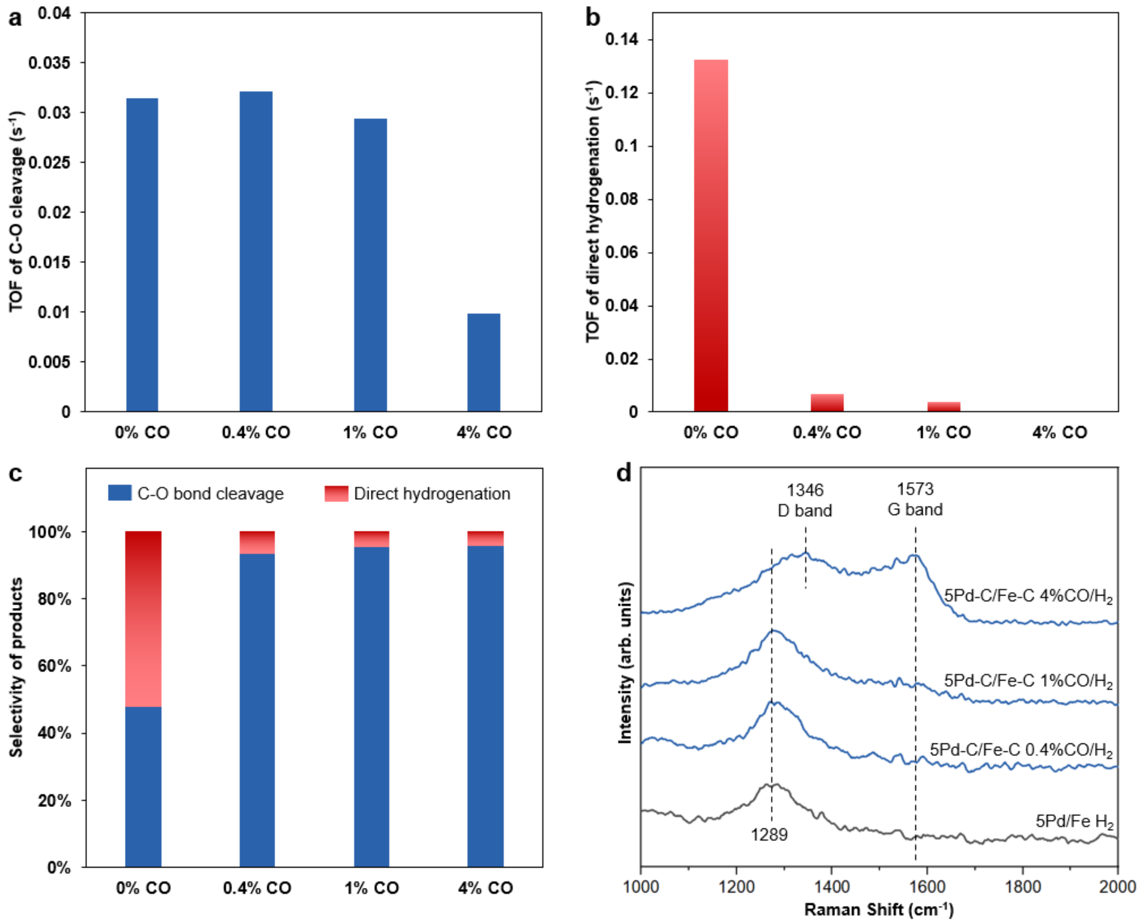

Supplementary Fig. 8 Investigation of 5Pd-C/Fe-C synthesized with different CO concentrations. a-b, TOFs of C-O bond cleavage (a, based on Fe) and direct hydrodeoxygenation (b, based on Pd) over catalysts synthesized with different CO concentrations (The data was derived at conversion < 20%). c, Selectivity of different categories of products at ~10% conversion. Reaction condition: 0.03 g catalyst, 50 mL $C_{16}H_{34}$, 1.08 g DPE, 250 °C, 5.6 MPa H_2. d, Raman spectra of catalysts to study the doped carbon species.

Supplementary Note 2 The possible role of graphitic carbon over catalyst in the conversion of DPE

To study the structure of the effective carbon species on the 5Pd-C/Fe-C, two more samples (i.e., 5Pd-C/Fe-C synthesized with 1 vol.% CO/H_2 and 4 vol.% CO/H_2) were prepared and evaluated in the hydrotreating of DPE, as shown in Supplementary Fig. S8a-c. The sample synthesized with 1 vol.% CO/H_2 showed similar catalytic behavior as that with 0.4 vol.% CO/H_2 (i.e., 5Pd-C/Fe-C presented in the main text). However, further increase of CO concentration significantly decreased the activity, though the high selectivity of C-O bond cleavage was maintained.

Raman spectroscopy was employed as a nondestructive tool with high resolution to study the carbon species[14,15]. Fig. S8d displays the Raman spectra of catalysts measured under visible excitation. The peak at ~1289 cm^{-1} are attributed to iron oxide[16] due to the surface oxidation when it's exposed to O_2. In the region of 1300 – 1600 cm^{-1}, the typical D and G bands, due to breathing modes of rings and bond stretching of pairs of *sp*^{2} carbon atoms respectively[17], gradually grow with the increase of CO concentration during synthesis, indicating the accumulation of graphitic carbon species. This is accompanied with the decrease of activity for the 4 vol.% CO-related catalyst that displays a pronounced D and G bands. It implies that the effective carbon species to simultaneously tune the hydrogenation chemistry and maintain the enhancement of Fe for C-O bond cleavage, does not likely have a graphitic structure.

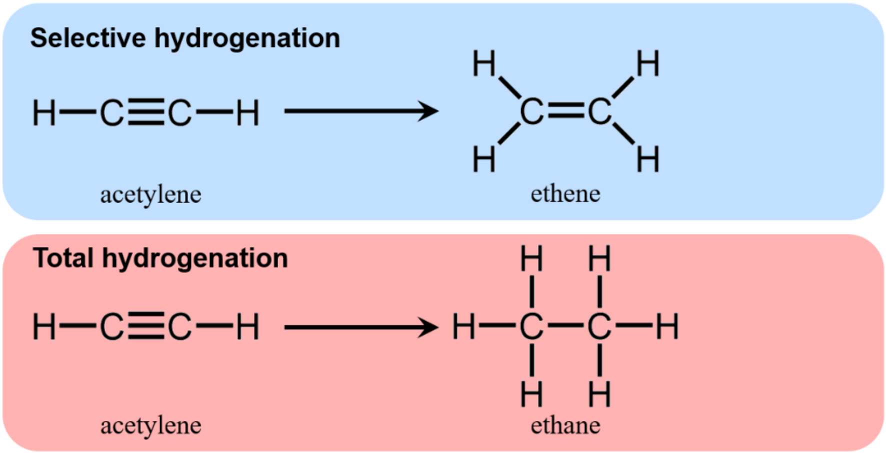

Supplementary Fig. 9 Different reaction pathways and products in the hydrogenation of acetylene.

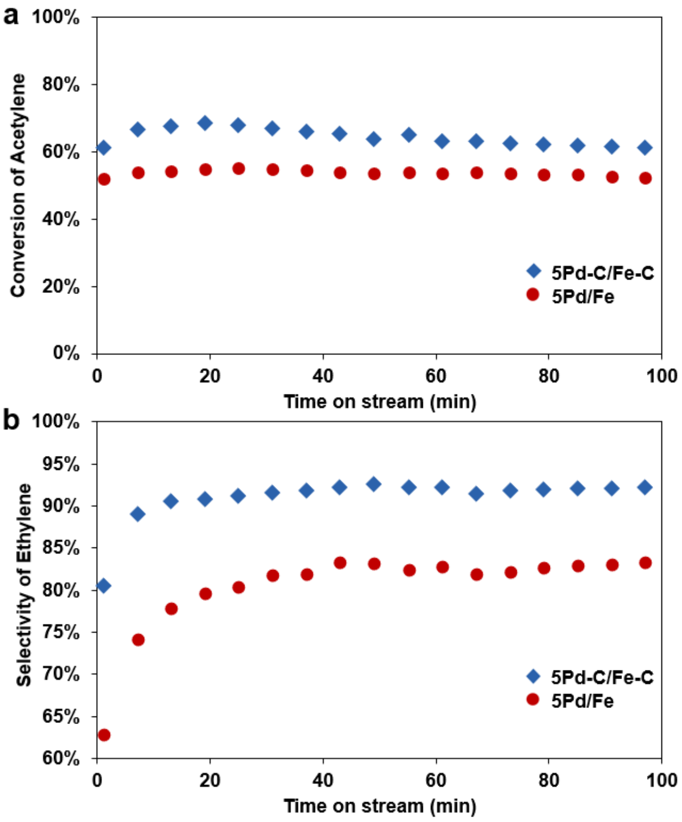

Supplementary Fig. 10 Performance of catalysts for hydrogenation of acetylene: conversion (a) and ethylene selectivity (b). Reaction condition: 0.03 g catalyst, 4% C_2H_2-8% H_2 balanced with N_2, GHSV=120,000 mL/(g*h), 150 °C, 0.1 MPa.

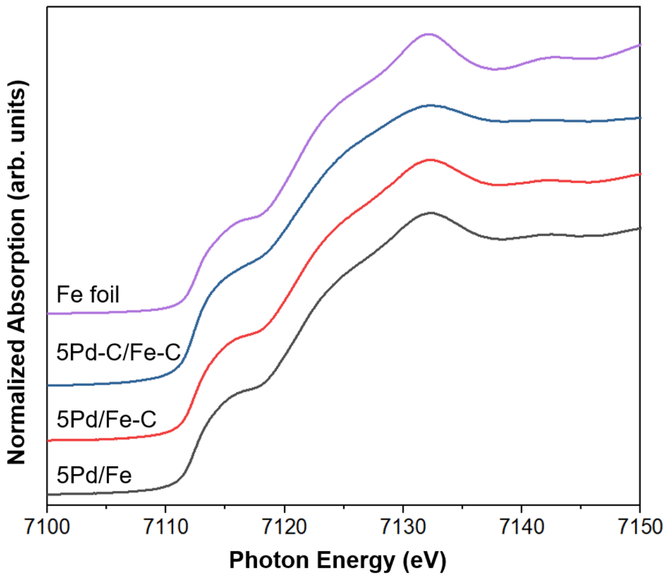

Supplementary Fig. 11 Normalized Fe K edge XANES of catalysts. The Fe foil was measured as reference.

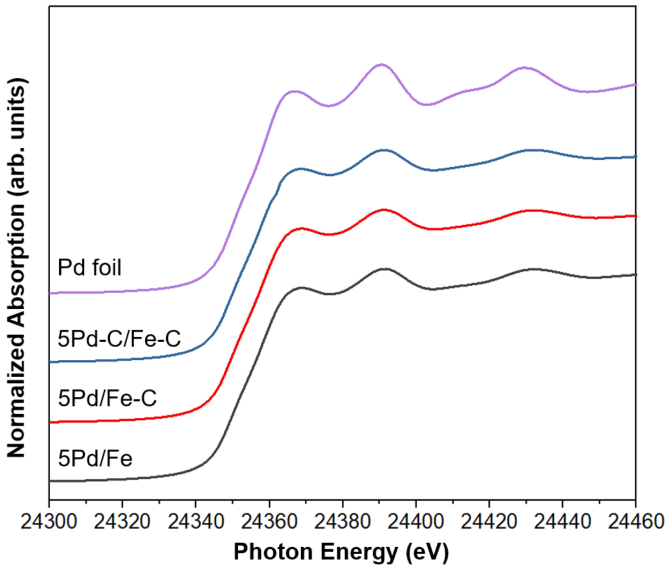

Supplementary Fig. 12 Pd K edge XANES of catalysts. The Pd foil was used as reference.

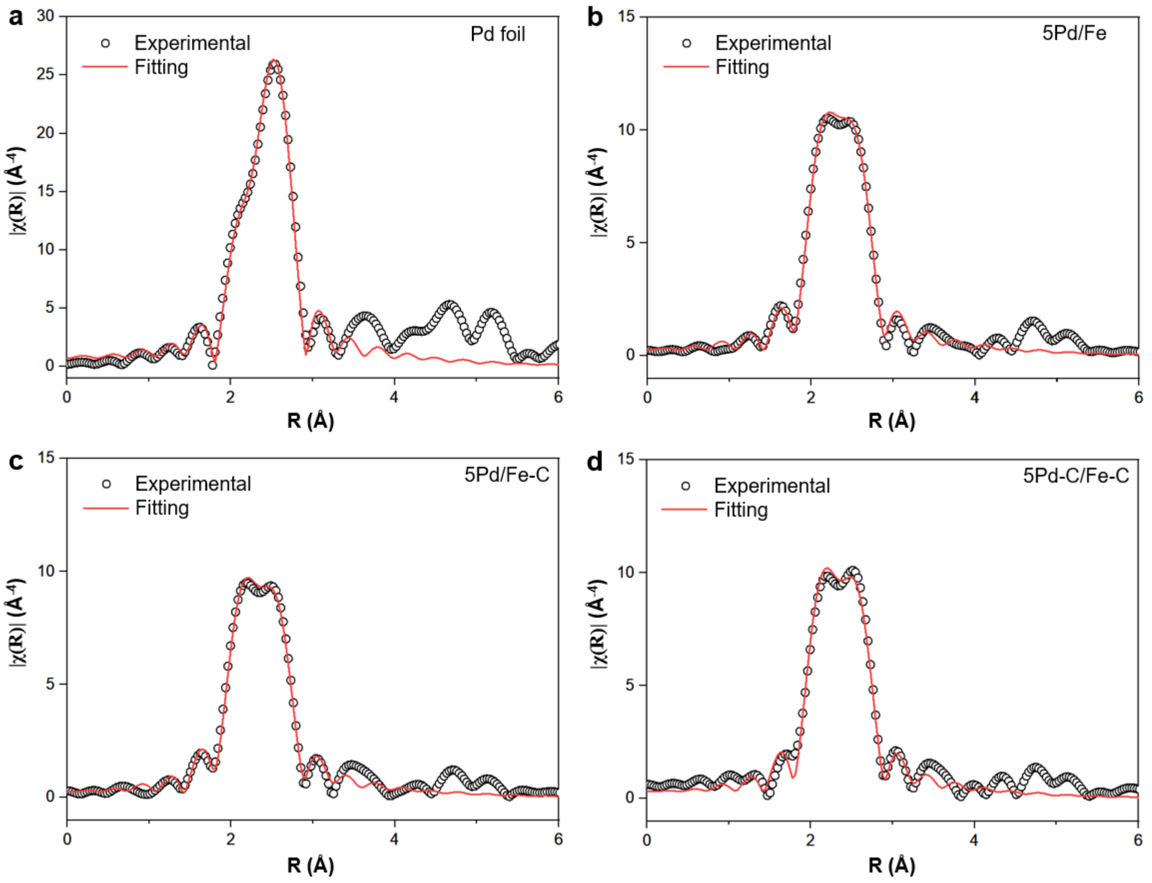

Supplementary Fig. 13 k^3-weighted Pd K edge EXAFS spectra and fitting results in R space for Pd foil (a), 5Pd/Fe (b), 5Pd/Fe-C (c) and 5Pd-C/Fe-C (d). The Pd foil was used as reference.

Supplementary Note 3

In the spectra of Fe K edge (Supplementary Fig. S11), all the catalysts show the position of the absorption edge, the intensities of the pre-edge and white line features that resemble to the standard Fe foil. No obvious absorption character related to carbide Fe[18] or oxidized Fe[19] is observed. It indicates the bulk Fe is in a metallic state for all the catalysts.

In the spectra of Pd K edge (Supplementary Fig. S12), the catalysts also display comparable absorption feature as metallic Pd foil, in contrast to oxidized Pd[20,21]. It suggests that Pd species is also in the metallic state. The Fourier transform was conducted for Pd foil and catalysts, as shown in Supplementary Fig. S13. The catalysts display different peak shapes and lower intensities,

compared with the Pd foil, which are likely due to the much smaller particle in the catalysts that lowers the total coordination number[21]. To further reveal the status of Pd, the curve fitting was then performed within acceptable Debye-Waller and R factors. The fitting results of EXAFS spectra (Supplementary Fig. S13 and Table S2) indicates two scattering paths that are attributed to Pd-Fe and Pd−Pd[22]. The coordination numbers (C.N.) for the three catalysts are comparable, suggesting all the catalysts share similar structure. Moreover, the C.N. for Pd-Pd (7-8) is much larger than that for Pd-Fe (1-2), indicating Pd mainly exists in a structure of nanoparticle other than the Pd-Fe alloy. The low Pd-Fe C.N. may be mainly contributed by the site anchoring Pd particle on Fe. Combining with other characterizations, it suggests the three catalysts share same structure, i.e., metallic Pd nanoparticles of similar size supported on bulk metallic Fe.

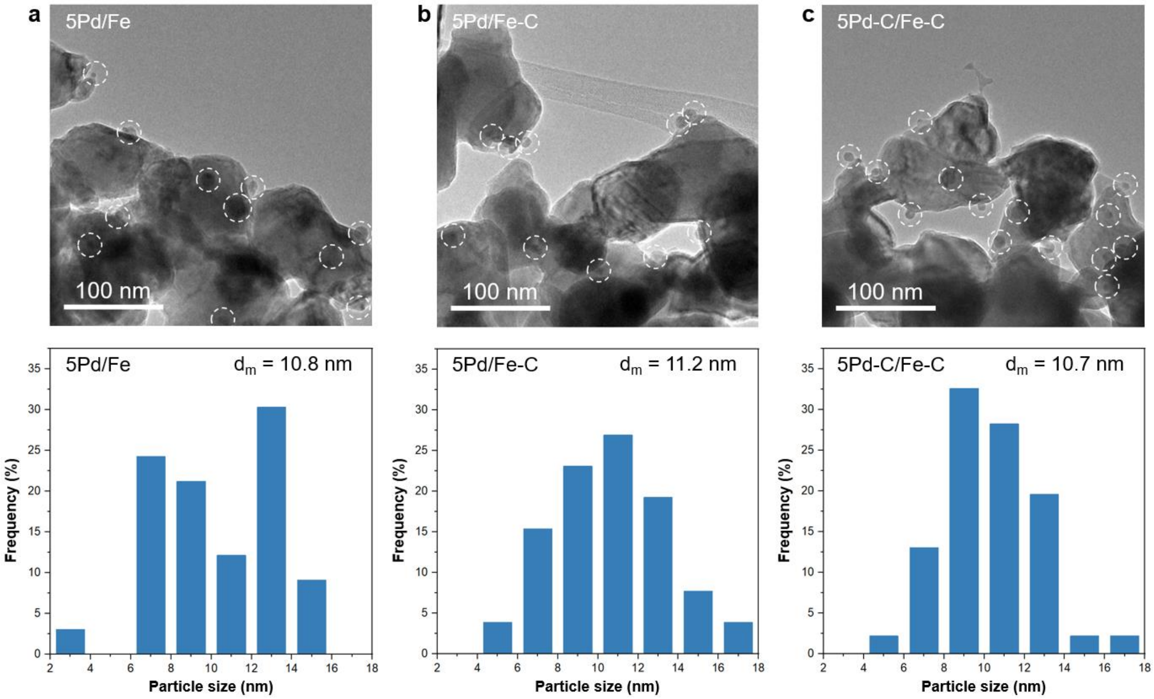

Supplementary Fig. 14 TEM images and Pd particle size distributions of 5Pd/Fe (a), 5Pd/Fe-C (b) and 5Pd-C/Fe-C (c). The analyses are based on 80–120 particles per sample.

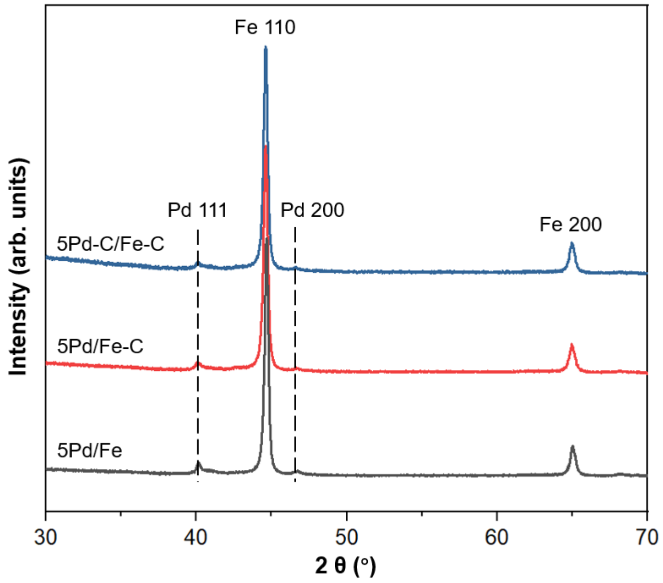

Supplementary Fig. 15 XRD patterns of as-synthesized 5Pd/Fe, 5Pd/Fe-C and 5Pd-C/Fe-C.

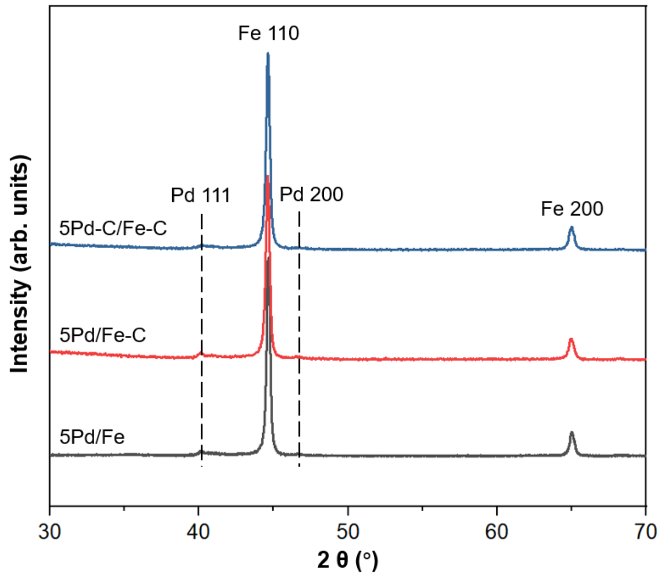

Supplementary Fig. 16 XRD patterns of spent catalysts collected after hydrotreating reaction.

Supplementary Note 4

The structure of catalysts with 5 wt.% Pd were studied with XRD. All the catalysts display patterns containing peaks at 44.7 ° and 65.0 °, that can be indexed to 110 and 200 diffractions of metallic Fe with body-centered cubic crystal structure (JCPDS no. 06-0696). The main diffraction peak corresponds to (110) facet, that is aligned with the HRTEM showing the support has a lattice fringe of 0.21 nm (Fig. 2a).[23] No diffractions of carbide of Fe[24,25] can be detected. Besides the peaks attributed to Fe, others with low intensity also appear at 40.1 ° and 46.7 °, which are the 111 and 200 diffractions of metallic Pd with face-centered cubic crystal structure (JCPDS no. 46-1043). The formation of interstitial carbide of Pd has been reported to expand the lattice and lower the diffraction angle about 1 °.[26] The absence of these peaks suggests the formation of Pd carbide in the 5Pd/Fe-C and 5Pd-C/Fe-C, if any, is negligible, which is aligned with the previous report showing its instability in H_2 above 150 °C.[26]

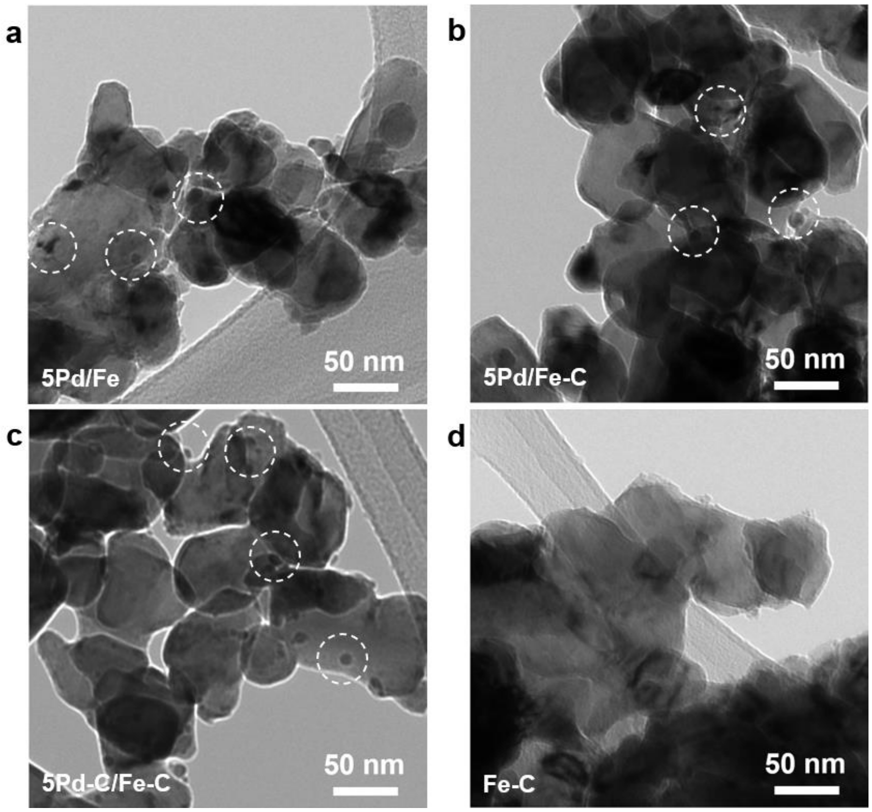

Supplementary Fig. 17 TEM images of spent catalysts after 4-h time-on-stream reaction. a, 5Pd/Fe; b, 5Pd/Fe-C; c, 5Pd-C/Fe-C; d, Fe-C.

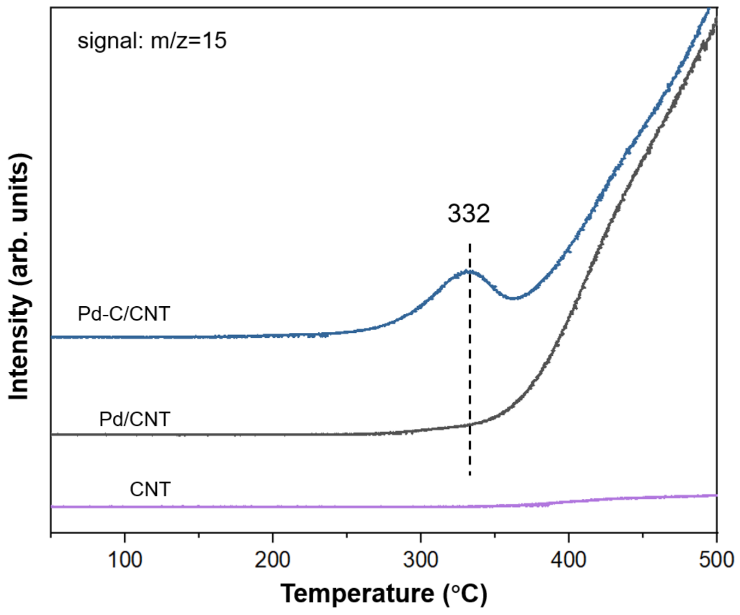

Supplementary Fig. 18 H_2-TPRea of Pd-C/CNT, Pd/CNT and CNT support.

Supplementary Note 5 H_2-TPRea profiles of Pd supported on CNT and bare Pd. Carbon ligand deposition was attempted using same procedure as 5Pd-C/Fe-C.

In Supplementary Fig. S18, the sample with carbon ligand obtained by the same synthesis procedure as 5Pd-C/Fe-C shows the methane formation peak at 332 °C, while the sample without carbon ligand shows no methane formation in the same region. This suggests the carbon ligand on Pd can react with H_2 at 332 °C. The methane formation at higher temperature (> 400 °C) is likely due to that Pd catalyzed the reaction between C support and H_2.

We also tried the same procedure as for 5Pd-C/Fe-C attempting to deposit the carbon ligand on Pd microparticle. The difference profile (signal of sample prepared with CO/H_2 – signal of sample prepared with UHP H_2) could clearly reflect the character/amount of deposited carbon ligand. As shown in Supplementary Fig. S25b, Pd microparticle shows negligible carbon deposition, in contrast to nanoparticle on Pd/CNT. As a result, no obvious difference of catalytic performances was detected between samples prepared with and without CO. It suggests that, though the mechanism remains elusive, the large microparticle hardly form effective carbon on Pd. Therefore, Pd nanoparticle model[27,28], other than plain Pd facet, was used in the theoretical calculation studying the possible carbon ligands on Pd.

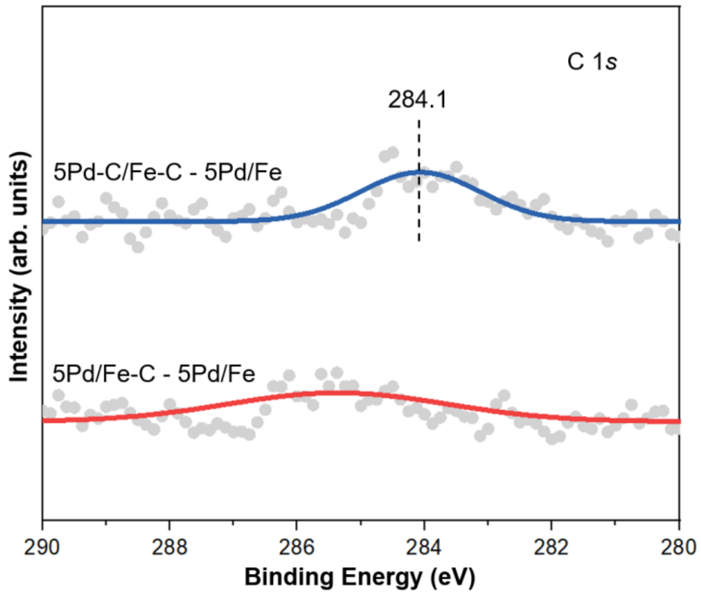

Supplementary Fig. 19 C 1*s* difference spectra of samples pseudo-*in situ* pretreated with 10% H_2/Ar at 250 °C for l h.

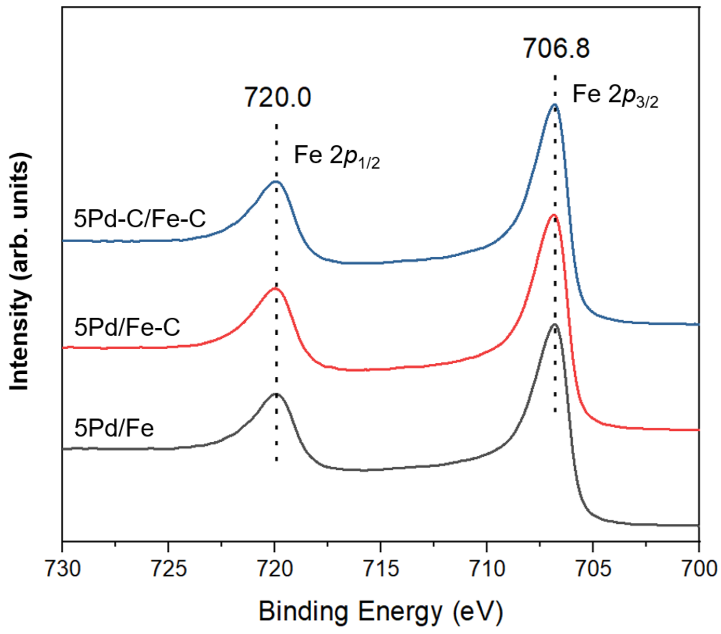

Supplementary Fig. 20 Fe 2*p* region of high energy resolution pseudo-*in situ* XPS of catalysts. All catalysts were pretreated with UHP H_2 at 250 °C for 0.5h prior to taking spectra.

Supplementary Note 6 Pseudo *in-situ* XPS characterization of the catalysts.

All the samples were pseudo-*in situ* pretreated with 10% H_2/Ar at 250 °C for l h. The pretreatment was to simulate the surface under hydrotreating condition. The difference spectra method for XPS study has been well developed to analyze the change of C species deposited on catalyst surface[29,30]. Subtraction was carried out without intensity normalization.

Since Fe is the main composite, that was least influenced by other components, in the catalysts and reduction at 250 °C was performed prior to the measurement, metallic Fe $2p_{3/2}$ at binding energy of 706.8 eV[31,32] was used for charge correction.

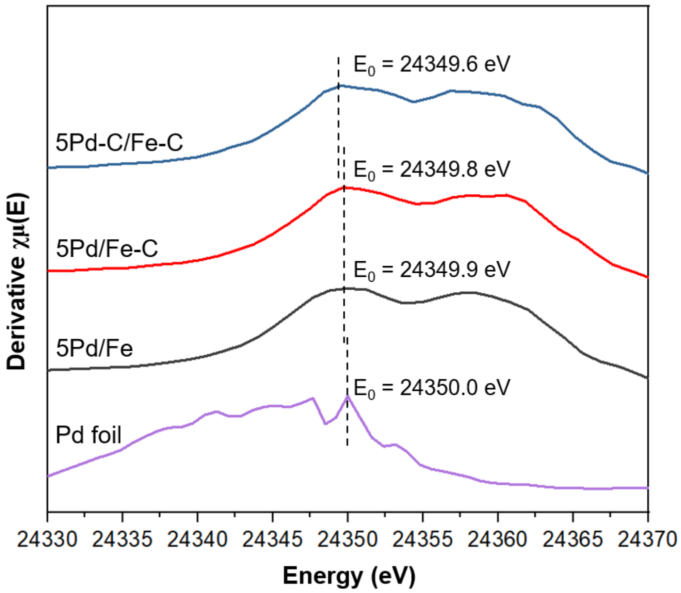

Supplementary Fig. 21 The first derivative of XANES of catalysts and Pd foil at Pd K-edge.

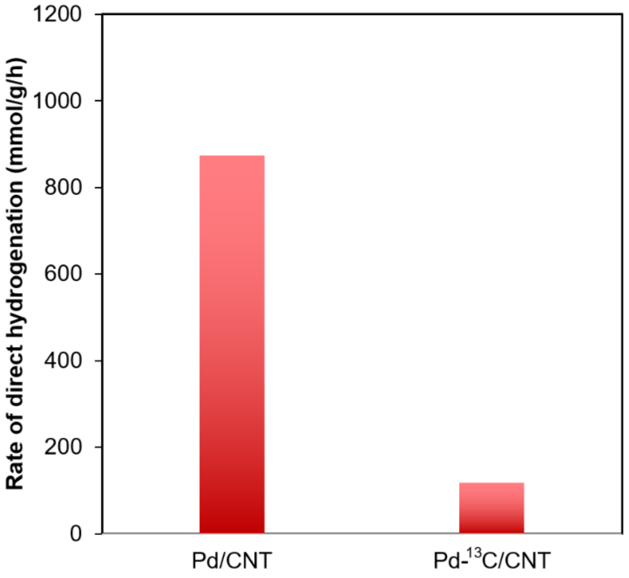

Supplementary Fig. 22 Catalytic performances of Pd/CNT and Pd-^{13}C/CNT. The amount of Pd is 5 wt.%. Reaction condition: 0.005 g catalyst, 50 mL $C_{16}H_{34}$, 1.08 g DPE, 250 °C, 5.6 MPa H_2.

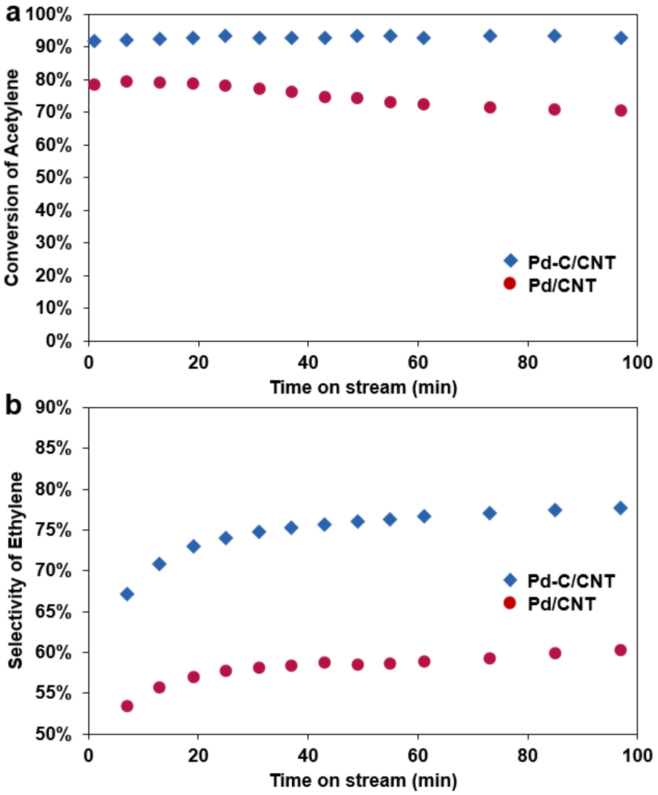

Supplementary Fig. 23 Performance of catalysts for hydrogenation of acetylene: conversion (a) and ethylene selectivity (b). Reaction condition: 0.012 g catalyst, 4% C_2H_2-8% H_2 balanced with N_2, GHSV=120,000 mL/(g*h), 150 °C, 0.1 MPa.

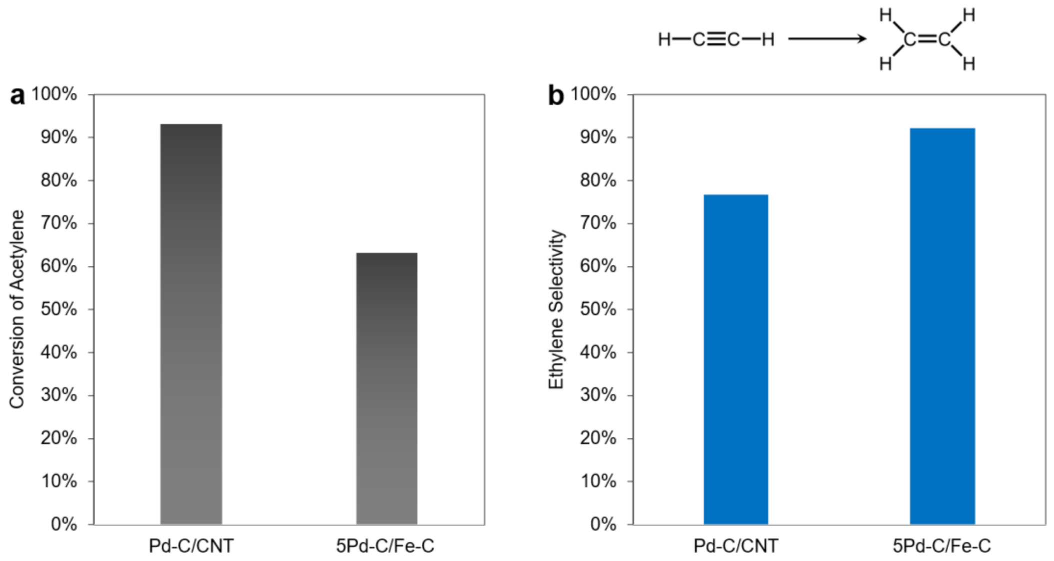

Supplementary Fig. 24 Performances of Pd-C/CNT (0.012 g), and 5Pd-C/Fe-C (0.03 g) for hydrogenation of acetylene: conversion (a) and ethylene selectivity (b). Reaction condition: 4% C_2H_2-8% H_2 balanced with N_2, GHSV=120,000 mL/(g*h), 150 °C, 0.1 MPa. The data was derived from Supplementary Fig. S10 and S23 at 60 min.

Considering the low density of CNT, the weight of Pd-C/CNT was lower than 5Pd-C/Fe-C to decrease the pressure drop. However, the same GHSV was maintained.

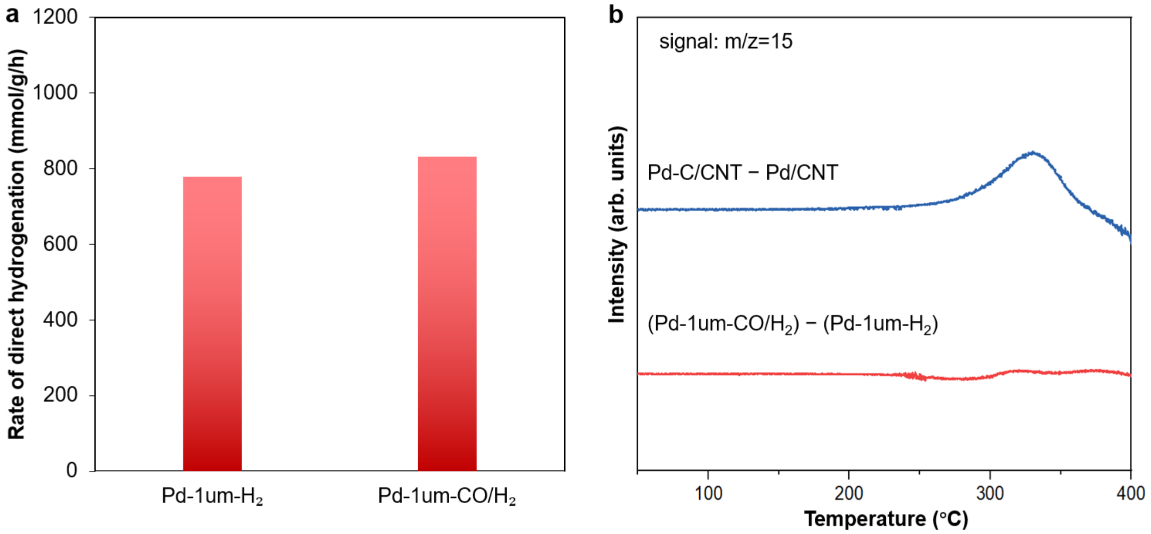

Supplementary Fig. 25 Catalytic performances (a) and the difference H_2-TPSR profile of Pd powders with ~1um size (b). Reaction condition: 0.005 g catalyst, 50 mL $C_{16}H_{34}$, 1.08 g DPE, 250 °C, 5.6 MPa H_2. In the H_2-TPSR experiment, the catalysts were *in situ* synthesized with H_2 and 0.4 vol.% CO/H_2. The H_2-TPSR profile of Pd/CNT series are shown as reference.

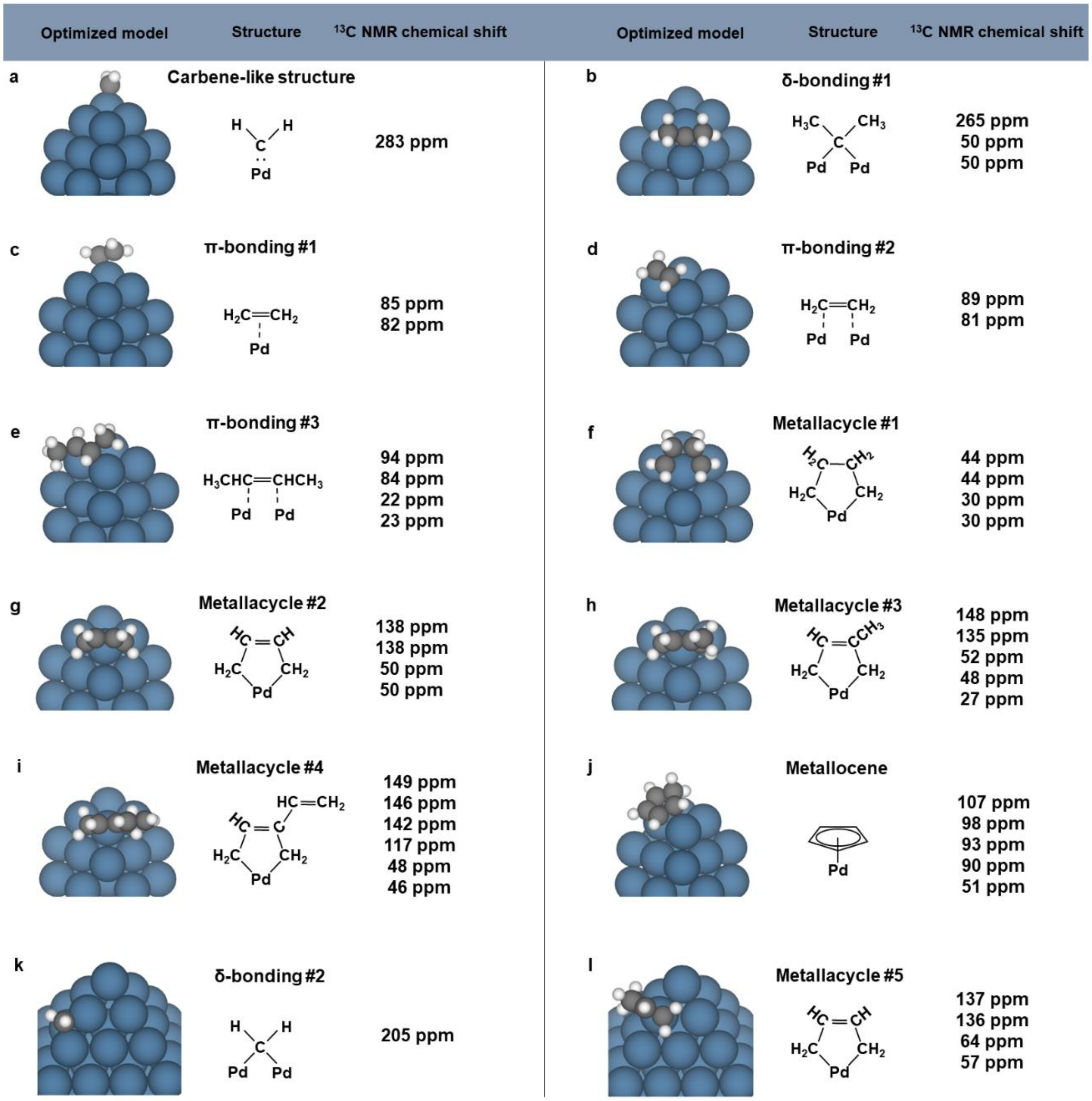

Supplementary Fig. 26 DFT clusters used to model different carbon species doped on Pd and the corresponding calculated ^{13}C NMR chemical shift. The carbon species analyzed includes carbene-like structure (a); carbonaceous species with δ bonding (b); carbonaceous species with π bonding (c-e); metallacycles (f-i); metallocene (j); carbonaceous species on larger Pd substrate (k-l). Color legend of atoms: Pd blue, C grey, and H white.

Supplementary Note 7 DFT modelling of catalyst structure and corresponding ^{13}C NMR chemical shift.

The reliability of GIAO method using B3LYP functional and Lanl2DZ basis set was examined by comparing the calculated and reported experimental values for chemical shifts of several molecules, e.g. hexene, pentene and butene[33]. The deviations for all the tested molecules were less than 5 ppm.

In the structural optimization, carbene was found unstable on Pd and preferred to form the bridge bonding with two adjacent Pd atoms at most of the studied sites of Pd particle. Moreover, at limited site to stabilize carbene coordinating Pd, the chemical shift is around 280 ppm that is far away from the experimental value. In fact, among all the calculated structures including alkene-type palladacycle[34], alkane-type palladacycle (linear δ bonding)[35], metallocene analog[36], alkene on Pd with δ-bonding[37], alkane-type carbon coordinating Pd with bridge configuration[38] and carbene-like structure[39], only alkene-type palladacycle (chemical shifts at around 140-150 and 50 ppm) fits well with the experimental result (chemical shifts at around 150 and 60 ppm).

It should also be noted that sp^2 carbon having δ bonding with Pd cluster is not stable, while the tested metallacycle structures shown in g, h and i keep the C=C bond away from Pd so that the hydrogenation of C=C bond is prevented.

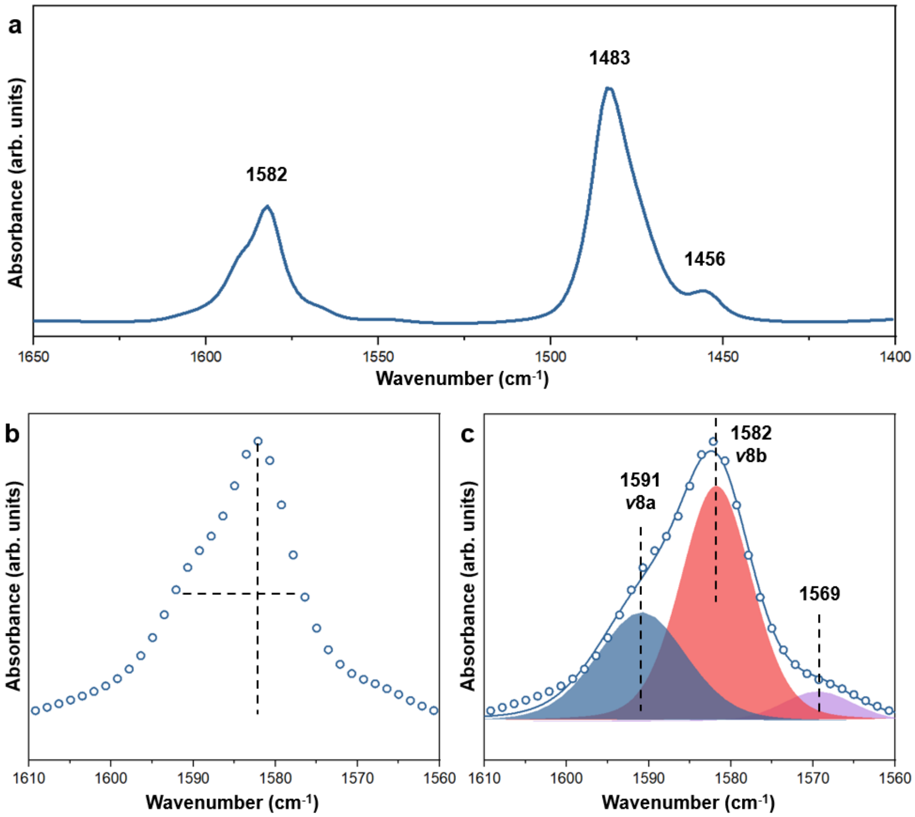

Supplementary Fig. 27 a, ATR-FTIR spectrum of DPE without catalyst. b, magnification of *v*8 band in the spectrum. c, the result of deconvolution to *v*8 band.

Supplementary Note 8

In Supplementary Fig. S27a, three peaks appear at 1582, 1483 and 1456 cm^{-1}, attributing to *v*8, *v*19 and C_{ring}-H bending vibration modes (Wilson's numbering)[40-42], respectively. Magnification of *v*8 band shows that it presents an asymmetric shape, as shown in Fig. S27b. We therefore deconvolute this peak as shown in Fig. S27c. The result indicates that the free DPE (i.e., the one without interaction with catalyst surface) has a character that the *v*8b has a much higher intensity than *v*8a. A different ratio of intensities for *v*8a and *v*8b indicates a perturbation towards the aromatic ring in DPE[43].

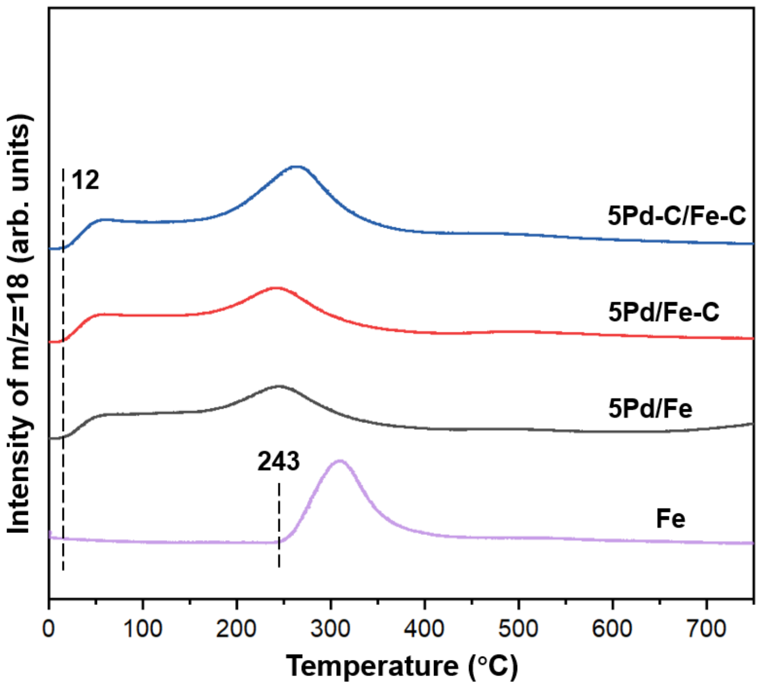

Supplementary Fig. 28 H_2-TPR of catalysts that are surface-oxidized. The catalysts were *in situ* synthesized.

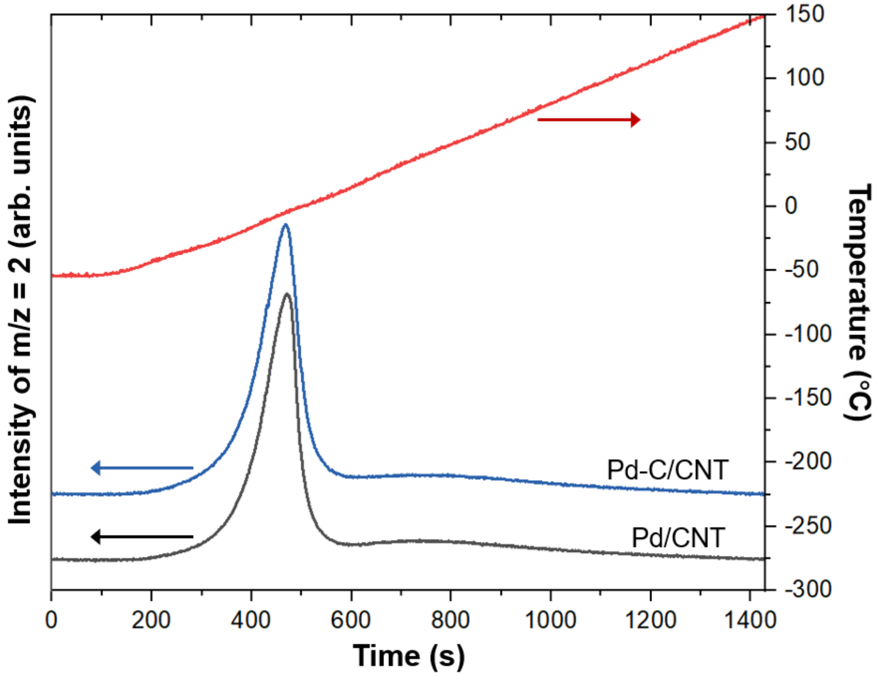

Supplementary Fig. 29 The H_2-TPD profile of Pd/CNT and Pd-C/CNT.

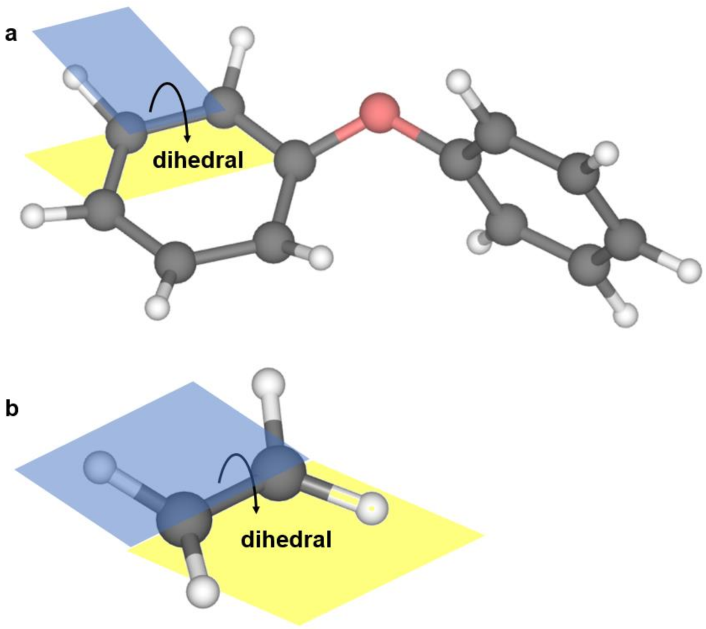

Supplementary Fig. 30 The way to measure the dihedral of C-H bonds to the plane formed by the adjacent three atoms. a, diphenyl ether; b, ethene.

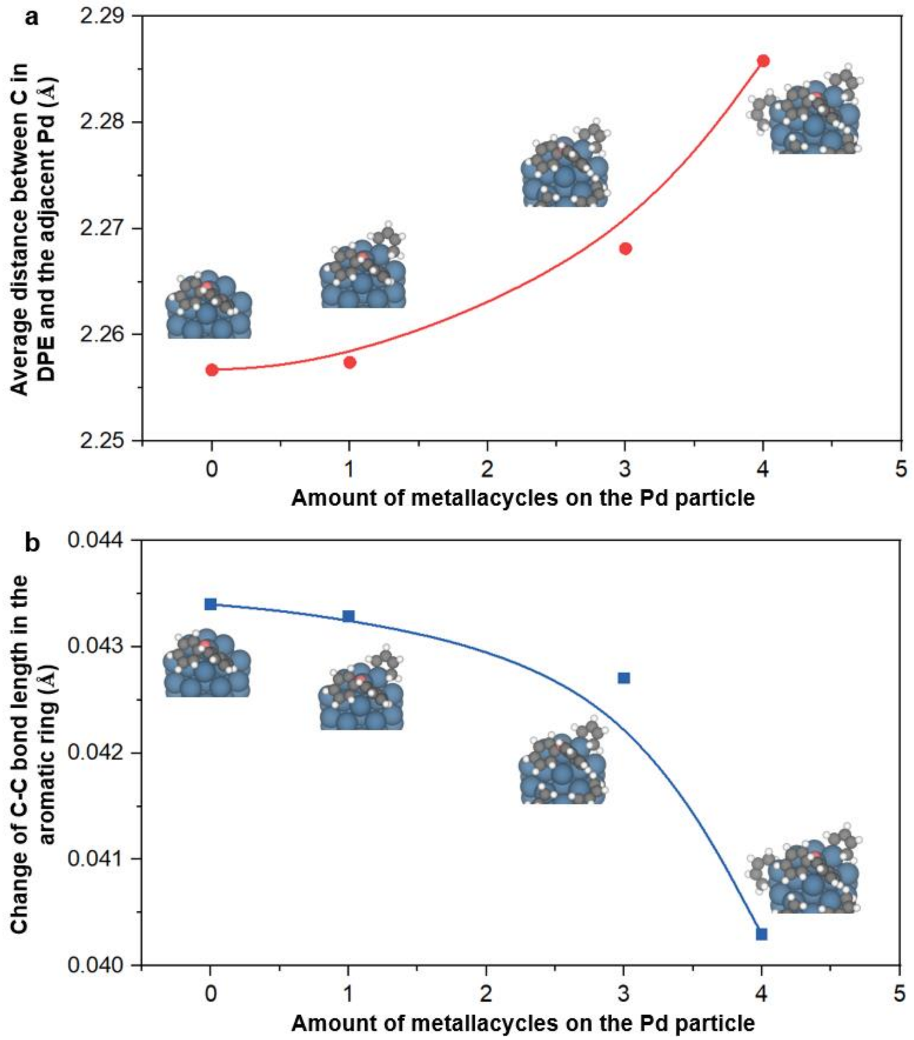

Supplementary Fig. 31 The influence on the interaction between Pd and aromatic ring by varying the concentration of metallacycle on Pd particle. a, the change of average distance between C in adsorbed aromatic ring and the adjacent Pd; b, the change of C-C bond length in the adsorbed aromatic ring.

Supplementary Table 1 Structural properties of the catalysts.

	Surface area (m^2/g)	Number of exposed Pd (mmol/g)[a]	Number of exposed Fe (mmol/g)	Dispersion of Pd (%)[a]	Particle size of Pd (nm)[a]	Particle size of Pd (nm)[b]
Fe	8.2	\[c]	0.19	\	\	\
0.1Pd/Fe	8.1	0.011	0.18	81.7	1.4	-
1Pd/Fe	8.0	0.027	0.15	20.2	5.5	-
5Pd/Fe	9.2	0.055	0.16	8.8	12.7	10.8
5Pd/Fe-C	9.2	0.052	0.16	8.3	13.4	11.2
5Pd-C/Fe-C	9.4	0.048	0.17	7.7	14.5	10.7

a) Determined by CO pulse chemisorption.

b) Determined by TEM.

c) The calculated value using the CO pulse chemisorption result is ~6×10^{-4} mmol/g, indicating that adsorption of CO on Fe is negligible for the calculation of numbers of exposed Fe and Pd (detailed procedure is shown in Supplementary Method).

Supplementary Table 2 Structural parameters determined by quantitative EXAFS curve-fitting.

Sample	Scattering pair	C.N.[a]	R(Å)[b]	δ^2(Å^{-2})[c]	R factor
Pd foil	Pd-Pd	12	2.74 ± 0.01	0.0057	0.0049
5Pd/Fe	Pd-Fe	1.3	2.55 ± 0.01	0.0083	0.0083
	Pd-Pd	7.2	2.71 ± 0.02	0.0071	
5Pd/Fe-C	Pd-Fe	1.9	2.49 ± 0.03	0.0115	0.0096
	Pd-Pd	7.3	2.73 ± 0.04	0.005	
5Pd-C/Fe-C	Pd-Fe	1.5	2.58 ± 0.02	0.0088	0.0143
	Pd-Pd	7.8	2.76 ± 0.03	0.0051	

a) CN: coordination number.

b) R: interatomic distances between Pd and surrounding atoms.

c) δ^2: Debye-Waller factor.

DFT Cluster Model Coordinates

Fig. 4d DPE/Pd

Pd	-0.429284	-0.033755	-1.309973
Pd	0.394295	1.942913	0.430378
Pd	4.090290	-1.623122	-1.212097
Pd	-2.677229	-1.991089	2.373622
Pd	-2.318047	1.911048	-0.473294
Pd	-1.386201	3.803959	1.296087
Pd	1.447571	-1.991785	-2.199385
Pd	2.191191	0.144466	-0.411940
Pd	-0.756735	-3.873831	1.534916
Pd	1.287303	2.052172	-2.286674
Pd	1.953195	4.001861	-0.443730
Pd	-2.034988	-2.289268	-0.394352
Pd	-1.147994	-0.066764	1.334015
Pd	2.467768	-3.704417	-0.217596
Pd	-0.599931	3.998278	-1.293568
Pd	3.121243	0.089050	-3.081442
Pd	3.865858	2.073865	-1.354341
Pd	-3.868038	-0.351277	0.384889
Pd	-3.034623	1.624248	2.221784
Pd	0.676025	-1.837125	0.533216
Pd	-1.863039	-0.038916	4.147359
Pd	4.896344	0.362381	0.491843
Pd	1.786863	-3.642650	2.495679
Pd	-0.305748	1.944245	3.181168
Pd	3.354218	-1.612556	1.531303
Pd	3.089398	2.175195	1.398851
Pd	0.028191	-1.771383	3.336190
Pd	1.552312	0.198186	2.377863
Pd	0.416160	-0.090276	-3.949606
Pd	-3.030042	-0.011491	-2.373255
Pd	-1.342767	1.960491	-3.118129
Pd	1.208036	3.971361	2.210793
Pd	-1.344793	-2.094093	-3.063865
Pd	-0.066943	-4.045470	-1.125063
C	-7.087895	2.279693	-0.254110
C	-6.267189	1.503311	-1.093450
C	-6.819488	0.375781	-1.726082
C	-8.167243	0.023756	-1.557127
C	-8.980034	0.813662	-0.720693
C	-8.441461	1.937802	-0.064120
H	-6.671021	3.154498	0.238647
H	-5.229037	1.774683	-1.269827
H	-8.563274	-0.840828	-2.082046
H	-10.026257	0.551564	-0.585285
H	-9.070316	2.544856	0.581674
O	-6.050566	-0.391794	-2.645581
C	-4.976505	-1.195785	-2.203172
C	-4.191233	-1.766340	-3.260009
C	-4.979307	-1.757681	-0.849398
C	-3.367619	-2.940854	-3.020695
H	-4.435275	-1.480794	-4.279321
C	-4.217640	-2.986915	-0.654243
H	-5.895975	-1.641512	-0.264297
C	-3.429026	-3.562923	-1.700828
H	-3.151778	-3.586498	-3.877723
H	-4.441330	-3.582542	0.226199
H	-3.076906	-4.584965	-1.595416

Fig. 4d DPE/Pd-C

Pd	-0.083835	0.132262	1.422657
Pd	0.282998	1.806098	-0.790124
Pd	-4.738319	0.517801	0.484164
Pd	1.623948	-3.986985	-1.252081
Pd	2.650496	0.879012	0.591797
Pd	2.798717	2.579249	-1.552151
Pd	-2.795340	-0.588956	2.097531
Pd	-2.194056	1.125697	-0.130229
Pd	-1.028414	-4.136128	-0.535808
Pd	-0.770093	2.926118	1.592055
Pd	-0.309509	4.411426	-0.751558
Pd	0.572262	-2.633511	1.138552
Pd	1.019560	-0.789019	-0.886057
Pd	-4.080654	-2.229646	0.224226
Pd	1.857430	3.582978	0.779092
Pd	-3.454873	2.174744	2.210650
Pd	-2.915004	3.681899	0.035516
Pd	3.215404	-1.932719	0.166336
Pd	3.977349	0.122533	-1.951804

Pd	-1.562353	-1.444539	-0.319194
Pd	2.551685	-1.987683	-2.779673
Pd	-4.345532	2.114378	-1.743941
Pd	-2.936191	-3.071373	-2.212948
Pd	1.433094	0.690630	-3.195343
Pd	-3.658785	-0.486000	-1.993227
Pd	-1.831375	2.741901	-2.484697
Pd	-0.318774	-2.403808	-2.703187
Pd	-1.152210	0.170925	-2.640628
Pd	-1.210011	1.112368	3.658656
Pd	2.059113	-0.836803	2.703291
Pd	1.394037	1.905411	2.972499
Pd	0.701978	3.333586	-3.155422
Pd	-0.654489	-1.670350	3.636552
Pd	-2.105636	-3.268930	1.827545
C	6.514052	3.382306	1.583280
C	5.839891	2.267042	2.112381
C	6.321692	1.682134	3.297025
C	7.444092	2.195657	3.964832
C	8.106028	3.315552	3.427483
C	7.647146	3.908491	2.234492
H	6.146752	3.841595	0.668766
H	4.952342	1.872696	1.620092
H	7.775155	1.724124	4.885150
H	8.975433	3.721294	3.938692
H	8.160952	4.772189	1.820995
O	5.636944	0.614912	3.933747
C	5.180698	-0.500010	3.222698
C	4.109171	-1.208915	3.837063
C	5.800248	-0.977454	2.057127
C	3.624599	-2.406123	3.227761
H	3.805519	-0.917358	4.838363
C	5.341234	-2.177637	1.457544
H	6.649095	-0.449962	1.635067
C	4.234811	-2.889788	2.005885
H	2.987766	-3.080101	3.795367
H	5.917737	-2.613585	0.646233
H	4.031810	-3.911665	1.694712
C	2.171433	-6.429816	-2.784987
H	2.067302	-7.235979	-3.515860
C	3.229500	-6.340424	-1.958734
H	4.051715	-7.060125	-1.981839
C	3.269359	-5.211797	-0.959916
H	3.127840	-5.551169	0.079627
H	4.175335	-4.593627	-1.026794
C	1.095653	-5.374538	-2.713043
H	0.993478	-4.800975	-3.647698
H	0.106482	-5.777838	-2.440537
C	6.590013	-0.389112	-3.194002
H	7.581044	-0.811429	-3.378265
C	6.017070	0.493879	-4.031513
H	6.514337	0.849835	-4.937533
C	4.632542	1.005050	-3.719154
H	4.595613	2.095980	-3.562515
H	3.890923	0.737475	-4.488548
C	5.834469	-0.799066	-1.955501
H	5.674863	-1.883087	-1.873638
H	6.283775	-0.418832	-1.024767
C	-1.168962	-3.774860	5.643798
H	-1.269970	-4.760105	6.106737
C	-1.574961	-2.648359	6.258276
H	-2.003996	-2.652104	7.263832
C	-1.450790	-1.336585	5.523781
H	-2.427231	-0.858336	5.344468
H	-0.773288	-0.621731	6.018811
C	-0.535314	-3.667547	4.279190
H	0.557738	-3.804293	4.299288
H	-0.946527	-4.398700	3.557119

Fig. 4d Ethene/Pd

Pd	0.123030	-0.001873	1.510427
Pd	-1.419024	1.371649	-0.308456
Pd	-1.572546	-4.099548	-0.359566
Pd	3.989137	1.406014	-0.865576
Pd	0.065680	2.902643	1.551741
Pd	-1.517581	4.119835	-0.356620
Pd	0.026800	-2.904645	1.549550
Pd	-1.438499	-1.353291	-0.309312
Pd	3.968443	-1.455820	-0.865771
Pd	-2.770575	0.017205	1.760862
Pd	-4.173000	1.460977	-0.141438

Pd	3.223239	-0.019582	1.435350
Pd	1.315562	1.337114	-0.500288
Pd	1.283638	-4.089268	-0.610519
Pd	-2.768362	2.870596	1.692553
Pd	-2.807548	-2.836135	1.691111
Pd	-4.193021	-1.406457	-0.141754
Pd	2.934206	2.845140	1.191696
Pd	1.338100	4.072583	-0.606756
Pd	1.295896	-1.353486	-0.502155
Pd	2.447237	2.689248	-2.746537
Pd	-3.004769	-2.692934	-2.253916
Pd	2.410391	-2.718134	-2.748654
Pd	-0.260404	2.690736	-2.508038
Pd	-0.297309	-2.684769	-2.510240
Pd	-2.956405	0.019867	-2.277815
Pd	2.411914	-0.014512	-2.783926
Pd	-0.252413	0.002791	-2.504472
Pd	-1.168582	-1.387791	3.543897
Pd	1.619062	1.395220	3.306055
Pd	-1.148250	1.399269	3.543959
Pd	-2.967458	2.733612	-2.251717
Pd	1.597708	-1.417505	3.306400
Pd	2.893688	-2.882187	1.189700
C	5.076091	0.655460	2.500197
H	4.760094	1.228898	3.369229
H	5.684556	1.187688	1.771705
C	5.045910	-0.749859	2.521766
H	5.630460	-1.329641	1.810168
H	4.705713	-1.282309	3.407593

Fig. 4d Ethene/Pd-C

Pd	-0.373528	-0.289885	1.475811
Pd	-0.010809	2.051651	-0.062273
Pd	-4.232959	-1.118184	-1.170847
Pd	3.623601	-2.474872	-0.865542
Pd	1.926693	1.591563	1.985983
Pd	2.062782	3.805793	0.305456
Pd	-2.658003	-1.933692	0.917560
Pd	-2.065484	0.422101	-0.594226
Pd	1.197537	-3.778899	-1.333070
Pd	-2.140260	2.051571	1.753565
Pd	-1.622876	4.227588	0.069912
Pd	1.557747	-2.608850	1.204671
Pd	1.654831	-0.063986	-0.278102
Pd	-2.476603	-3.270991	-1.562013
Pd	0.095773	3.755283	2.189436
Pd	-4.357005	0.310868	1.105491
Pd	-3.822978	2.491563	-0.365824
Pd	3.639675	-0.598006	1.397339
Pd	4.119669	1.947327	0.225354
Pd	-0.427839	-1.666123	-0.885091
Pd	3.966984	0.051607	-1.706110
Pd	-3.857550	1.109237	-2.739410
Pd	-0.358746	-2.944262	-3.448603
Pd	2.124680	2.181827	-1.972809
Pd	-2.095291	-0.919110	-3.109171
Pd	-1.690951	2.697256	-2.296583
Pd	1.753123	-1.387658	-2.750197
Pd	0.000347	0.645974	-2.564160
Pd	-2.478838	-0.254639	3.174678
Pd	1.441717	-0.773721	3.315749
Pd	-0.287286	1.406463	3.687907
Pd	0.409273	4.285265	-1.830734
Pd	-0.933223	-2.709883	2.993121
Pd	-0.820331	-4.068196	0.502664
C	6.078552	-3.253532	-2.279040
H	6.771877	-3.479353	-3.093242
C	6.486757	-3.122897	-1.002476
H	7.531681	-3.245685	-0.707120
C	5.458769	-2.795963	0.051881
H	5.266026	-3.630997	0.745014
H	5.712069	-1.896854	0.640240
C	4.611880	-3.090877	-2.592590
H	4.415360	-2.386338	-3.413801
H	4.108760	-4.044828	-2.820865
C	6.663356	2.771718	1.363621
H	7.567343	2.758720	1.978099
C	6.183437	3.909292	0.823088
H	6.668684	4.875035	0.985516
C	4.950978	3.851471	-0.045045
H	4.239747	4.670949	0.176460

H	5.173037	3.874701	-1.121853
C	5.934964	1.473729	1.114354
H	6.465100	0.803219	0.423212
H	5.696320	0.926674	2.043557
C	-2.160084	-4.956571	4.445258
H	-2.493268	-5.970108	4.682085
C	-2.607067	-3.876953	5.113502
H	-3.339177	-3.949428	5.921345
C	-2.055833	-2.523400	4.741462
H	-2.873289	-1.770045	4.654786
H	-1.315393	-2.142815	5.462138
C	-1.158590	-4.751226	3.335921
H	-0.153717	-5.121091	3.593649
H	-1.478373	-5.247126	2.393643
C	2.682402	-3.827624	2.715338
H	2.115657	-3.752281	3.640896
H	3.693234	-3.427671	2.740735
C	2.283119	-4.710151	1.702314
H	2.972126	-5.001452	0.913640
H	1.402183	-5.336535	1.819309

Fig. S23a

Pd	-0.179291	1.659765	1.629009
Pd	0.032359	-1.944023	1.498151
Pd	3.153584	1.966174	-1.191861
Pd	-2.594151	1.688570	-1.422910
Pd	-1.521047	-0.467527	2.950563
Pd	1.305815	3.652071	0.314793
Pd	1.478385	-0.008572	0.003536
Pd	-4.605476	0.119105	-0.440168
Pd	1.329070	-0.116689	2.989465
Pd	4.367042	0.022146	0.142239
Pd	-1.539214	3.458701	0.268194
Pd	-1.285874	-0.064358	-0.002110
Pd	2.774433	1.715409	1.549529
Pd	2.907424	-1.854286	1.417982
Pd	-3.111475	1.357671	1.481997
Pd	-3.308199	-1.560791	1.204340
Pd	0.278402	1.926703	-1.383866
Pd	-2.716537	-1.408173	-1.693016
Pd	3.147191	-1.828634	-1.329080
Pd	-1.530466	-3.293639	-0.057522
Pd	1.997656	0.126047	-2.809541
Pd	1.290408	-3.639085	-0.089116
Pd	-0.843889	0.234417	-3.077027
Pd	0.253708	-1.732494	-1.665291
C	-6.087168	-0.040170	-1.572822
H	-6.052891	-0.325768	-2.631723
H	-7.097475	0.175429	-1.192748

Fig. S23b

Pd	-0.303406	1.906261	1.291772
Pd	-0.302028	-1.907037	1.290702
Pd	3.233884	1.905197	-1.053785
Pd	-2.651199	1.477787	-1.870776
Pd	-1.875629	-0.001509	2.718058
Pd	1.329674	3.748119	-0.044976
Pd	1.396052	0.000177	0.025046
Pd	-4.425959	-0.000777	-0.280670
Pd	0.871375	-0.001202	2.937288
Pd	4.186547	0.001300	0.573154
Pd	-1.451067	3.524954	-0.595018
Pd	-1.381363	-0.000532	-0.188020
Pd	2.513018	1.867615	1.635625
Pd	2.515112	-1.867635	1.634495
Pd	-3.064920	1.939180	1.076295
Pd	-3.063875	-1.941836	1.074599
Pd	0.412670	1.824651	-1.659489
Pd	-2.651172	-1.477326	-1.872210
Pd	3.236069	-1.902256	-1.054641
Pd	-1.448488	-3.524162	-0.598509
Pd	2.233748	0.000970	-2.709415
Pd	1.332173	-3.746481	-0.048096
Pd	-0.698815	0.001100	-3.219845
Pd	0.413913	-1.823141	-1.661748
C	-0.631263	-0.005108	4.305333
C	-0.694872	1.271668	5.134699
H	-1.618486	1.283196	5.738938
H	0.151206	1.303718	5.842892
H	-0.667923	2.177717	4.524179

C	-0.694286	-1.285394	5.129285
H	0.152950	-1.320973	5.835917
H	-1.616929	-1.299089	5.734952
H	-0.668685	-2.188782	4.514777

Fig. S23c

Pd	0.403596	1.864365	-1.467333
Pd	0.324120	-1.829640	-1.488760
Pd	-2.938253	1.898207	1.294126
Pd	2.744911	1.691756	1.604811
Pd	2.052267	-0.002936	-2.744883
Pd	-1.213714	3.738031	-0.081083
Pd	-1.312256	0.028147	-0.050302
Pd	4.378363	-0.083415	0.293156
Pd	-0.878408	0.053482	-3.001313
Pd	-4.225532	0.107538	-0.323418
Pd	1.562656	3.660355	0.159096
Pd	1.468279	-0.021968	0.071937
Pd	-2.478224	1.857389	-1.533054
Pd	-2.539257	-1.693344	-1.625312
Pd	3.266551	1.812438	-1.175826
Pd	3.170200	-1.912435	-1.201014
Pd	-0.174376	1.850495	1.501024
Pd	2.714235	-1.811450	1.580348
Pd	-3.090296	-1.836619	1.135571
Pd	1.370258	-3.681162	0.163207
Pd	-1.830905	-0.111682	2.777213
Pd	-1.386204	-3.689114	-0.163073
Pd	1.056838	-0.031686	2.995273
Pd	-0.237275	-1.876788	1.492173
C	-6.242202	-0.487655	-1.042842
H	-6.239236	-0.344329	-2.121739
C	-6.382784	0.604234	-0.170159
H	-6.660020	0.455201	0.871680
H	-6.491814	1.616829	-0.554636
H	-6.407492	-1.505573	-0.695144

Fig. S23d

Pd	0.713562	1.952117	-1.282482
Pd	0.343157	-1.759294	-1.630784
Pd	-2.899036	2.273171	0.840570
Pd	2.549459	0.984267	2.189443
Pd	2.277942	-0.013211	-2.684244
Pd	-0.730410	3.878038	0.134692
Pd	-1.285573	0.179473	-0.246473
Pd	4.295011	-0.267202	0.465274
Pd	-0.621369	0.374034	-3.065288
Pd	-3.960377	0.580397	-0.965490
Pd	2.006331	3.193662	0.788189
Pd	1.424751	-0.127816	0.126116
Pd	-2.066573	2.320723	-1.737876
Pd	-2.494661	-1.389378	-2.092556
Pd	3.412762	1.708947	-1.043958
Pd	3.109773	-2.019415	-1.159981
Pd	-0.237632	1.675834	1.683933
Pd	2.391081	-1.872750	1.772181
Pd	-3.477010	-1.625532	0.556257
Pd	1.154469	-3.656689	0.147124
Pd	-2.465218	-0.015089	2.398531
Pd	-1.602301	-3.501009	-0.608510
Pd	0.348038	-0.449038	3.172010
Pd	-0.640392	-1.901738	1.245019
C	-4.708011	-1.870952	2.307524
H	-5.661329	-1.516483	1.904241
C	-4.081090	-1.087666	3.338025
H	-3.505791	-1.617155	4.103247
H	-4.603629	-0.189134	3.686705
H	-4.600753	-2.960590	2.354491

Fig. S23e

Pd	0.995649	1.956477	-1.241968
Pd	0.376068	-1.718051	-1.684960
Pd	-2.572095	2.579920	0.695619
Pd	2.514973	0.760988	2.364604
Pd	2.523394	-0.131945	-2.566240
Pd	-0.304596	4.002392	0.126817
Pd	-1.189777	0.315487	-0.385323
Pd	4.287677	-0.612414	0.781215
Pd	-0.292085	0.494294	-3.143360
Pd	-3.814485	0.946329	-1.081694
Pd	2.261204	3.049136	0.987192

Pd	1.460229	-0.205984	0.174977
Pd	-1.678613	2.516852	-1.887110
Pd	-2.393839	-1.098229	-2.334593
Pd	3.616858	1.465776	-0.757080
Pd	3.079426	-2.225842	-1.004548
Pd	-0.112294	1.703571	1.646383
Pd	2.144110	-2.057010	1.844999
Pd	-3.522112	-1.423685	0.197588
Pd	0.934021	-3.729471	0.058515
Pd	-2.557346	0.144379	2.153636
Pd	-1.727585	-3.371484	-0.930359
Pd	0.105706	-0.525590	3.096896
Pd	-0.818873	-1.879928	1.057323
C	-4.776129	-1.719322	1.907252
H	-5.715035	-1.388953	1.439942
C	-4.275673	-0.827427	2.944532
H	-3.760078	-1.337952	3.770866
C	-5.115881	0.377874	3.372739
H	-4.516754	1.155760	3.866866
H	-5.634099	0.830549	2.519641
H	-5.877261	0.054997	4.103070
C	-4.672098	-3.233278	2.112465
H	-3.714193	-3.511473	2.566726
H	-5.478703	-3.572428	2.784824
H	-4.783432	-3.792054	1.173749

Fig. S23f

Pd	0.672328	1.850067	-1.592993
Pd	0.671605	-1.849917	-1.593458
Pd	-3.104612	1.923493	0.620414
Pd	2.345042	1.857258	1.923442
Pd	2.618760	-0.000456	-2.405751
Pd	-1.300536	3.582468	-0.756556
Pd	-1.282973	0.000264	-0.409244
Pd	4.141970	-0.000506	1.110148
Pd	-0.202696	0.000701	-3.278312
Pd	-4.237467	-0.000307	-0.949339
Pd	1.399725	3.771886	0.115095
Pd	1.447454	-0.000138	0.168926
Pd	-2.286509	1.551860	-2.219674
Pd	-2.286253	-1.551056	-2.220111
Pd	3.406700	1.917799	-0.625241
Pd	3.406123	-1.918729	-0.625147
Pd	-0.391568	1.904151	1.192782
Pd	2.344705	-1.857789	1.923213
Pd	-3.104960	-1.923324	0.621074
Pd	1.398739	-3.772234	0.114594
Pd	-2.235004	0.000200	2.443670
Pd	-1.301740	-3.581951	-0.756554
Pd	0.495427	0.000040	2.920172
Pd	-0.391885	-1.903884	1.192445
C	-2.571254	1.324542	4.020851
C	-2.564710	-1.321782	4.024088
C	-3.804818	-0.781764	4.760126
C	-3.802862	0.779194	4.768058
H	-2.693543	-2.319526	3.588904
H	-3.828785	-1.186785	5.785740
H	-3.810527	1.173025	5.798157
H	-2.710170	2.319224	3.581663
H	-1.646866	-1.290066	4.622780
H	-1.649545	1.302934	4.613998
H	-4.711747	-1.143209	4.259650
H	-4.716192	1.148066	4.285018

Fig. S23g

Pd	-0.587272	-1.849046	-1.614154
Pd	-0.586837	1.848760	-1.614383
Pd	3.106059	-1.921033	0.744951
Pd	-2.399483	-1.848854	1.827086
Pd	-2.500814	-0.000230	-2.511054
Pd	1.347860	-3.581189	-0.691100
Pd	1.322982	-0.000137	-0.358659
Pd	-4.169087	0.000532	0.938816
Pd	0.363924	-0.000418	-3.260813
Pd	4.292693	-0.000184	-0.781891
Pd	-1.384586	-3.768022	0.067520
Pd	-1.434325	0.000065	0.108406
Pd	2.396358	-1.561142	-2.120772
Pd	2.396504	1.560233	-2.121187
Pd	-3.359567	-1.917972	-0.762619

Pd	-3.359035	1.918361	-0.763100
Pd	0.367900	-1.896736	1.212901
Pd	-2.399115	1.849544	1.826594
Pd	3.106588	1.920936	0.745041
Pd	-1.383872	3.768518	0.066648
Pd	2.167627	0.000204	2.550002
Pd	1.348788	3.580709	-0.691510
Pd	-0.583848	0.000230	2.904203
Pd	0.368186	1.896766	1.212541
C	2.336288	-1.402573	4.080090
C	2.332252	1.402971	4.080645
C	2.445094	0.673193	5.394940
C	2.447001	-0.673009	5.394679
H	3.248671	1.948010	3.799141
H	2.520821	1.256140	6.316305
H	2.524370	-1.256110	6.315811
H	3.254291	-1.944860	3.798432
H	1.475664	2.087399	4.024889
H	1.481542	-2.089241	4.023852

Fig. S23h

Pd	-0.648367	-1.593895	-1.881693
Pd	-0.731354	2.061946	-1.341086
Pd	3.167163	-1.898370	0.254133
Pd	-2.271425	-2.150992	1.607805
Pd	-2.647705	0.306456	-2.400933
Pd	1.372833	-3.378387	-1.326758
Pd	1.284583	0.108827	-0.462850
Pd	-4.125269	-0.244290	1.090601
Pd	0.170440	0.501052	-3.285740
Pd	4.232858	0.259078	-1.020814
Pd	-1.307890	-3.761171	-0.462278
Pd	-1.441837	-0.041374	0.134696
Pd	2.301628	-1.137886	-2.488166
Pd	2.231274	1.932982	-2.033066
Pd	-3.367235	-1.870140	-0.913633
Pd	-3.453966	1.924373	-0.351951
Pd	0.459377	-2.025346	0.856731
Pd	-2.355425	1.512687	2.149480
Pd	3.080092	1.904497	0.818008

Pd	-1.478812	3.694655	0.639333
Pd	2.278709	-0.287260	2.369002
Pd	1.211674	3.697752	-0.279372
Pd	-0.447802	-0.421108	2.851246
Pd	0.372923	1.731349	1.411902
C	2.530729	-1.873772	3.686693
C	2.456725	0.881211	4.082810
C	2.529677	-0.023051	5.284893
C	2.570449	-1.360030	5.109708
H	3.396486	1.419863	3.873668
H	2.546594	0.430725	6.279337
H	3.492144	-2.299771	3.352166
H	1.627419	1.599879	4.112940
H	1.733293	-2.608570	3.509476
C	2.648985	-2.381611	6.226410
H	3.540730	-3.016812	6.118215
H	2.690071	-1.900147	7.210635
H	1.776373	-3.051606	6.209055

Fig. S23i

Pd	0.665281	1.028984	-2.249771
Pd	0.911010	-2.327594	-0.724086
Pd	-3.256430	1.666714	-0.402545
Pd	2.094899	2.616318	0.998621
Pd	2.781631	-0.812043	-2.167084
Pd	-1.466991	2.764976	-2.272899
Pd	-1.240915	-0.339128	-0.476729
Pd	4.065668	0.759116	1.080850
Pd	0.019869	-1.414462	-3.049225
Pd	-4.149809	-0.820240	-1.050120
Pd	1.144840	3.536069	-1.461023
Pd	1.444765	0.138328	0.138193
Pd	-2.231412	0.238299	-2.794937
Pd	-2.018867	-2.577569	-1.512416
Pd	3.320347	1.727798	-1.315353
Pd	3.574999	-1.758451	0.270140
Pd	-0.590140	2.122247	0.229693
Pd	2.347211	-0.753091	2.528293
Pd	-2.996176	-1.829919	1.198478
Pd	1.651870	-3.312447	1.649684

Pd	-2.386042	0.755884	2.096243
Pd	-0.990289	-3.731552	0.688456
Pd	0.312833	1.177449	2.613660
Pd	-0.332139	-1.330086	1.795792
C	-2.803440	2.627627	2.896261
C	-2.514430	0.124374	4.076382
C	-2.599265	1.331386	4.963567
C	-2.775406	2.557936	4.409501
H	-3.459834	-0.443009	4.018552
H	-2.529110	1.196819	6.046787
H	-3.800249	2.858031	2.487783
H	-1.697608	-0.564294	4.324947
H	-2.068797	3.329267	2.480346
C	-2.920321	3.775917	5.223052
H	-2.874574	3.624850	6.303683
C	-3.104084	5.032018	4.746643
H	-3.156893	5.243723	3.680645
H	-3.207945	5.881378	5.416776

Fig. S23j

Pd	0.840046	2.013374	-1.270877
Pd	0.579693	-1.651902	-1.779931
Pd	-3.050864	1.876535	0.764297
Pd	2.581347	1.327769	2.114132
Pd	2.632464	0.204023	-2.465749
Pd	-1.036567	3.720262	0.124886
Pd	-1.223335	0.163033	-0.472027
Pd	4.270746	-0.415151	0.869798
Pd	-0.181128	0.490366	-3.203634
Pd	-4.037043	0.518601	-1.430790
Pd	1.670597	3.500411	0.780095
Pd	1.499932	-0.108277	0.163644
Pd	-1.988101	2.249843	-1.915191
Pd	-2.229199	-1.264745	-2.409501
Pd	3.579957	1.760027	-0.489742
Pd	3.270156	-1.995903	-1.018504
Pd	-0.279396	1.598313	1.576981
Pd	2.310480	-2.182283	1.618197
Pd	-3.311036	-1.268104	0.505691
Pd	1.089719	-3.779287	-0.237130
Pd	-2.120600	-0.009622	2.637489
Pd	-1.637960	-3.301493	-0.802751
Pd	0.679259	-0.457629	3.051861
Pd	-0.495295	-1.878557	1.085311
C	-5.192306	-0.480831	2.791439
H	-5.244020	0.497289	2.293814
H	-6.115840	-0.582610	3.389268
C	-5.062149	-1.655092	1.817781
H	-5.883734	-1.967433	1.180361
C	-4.011932	-2.507605	2.259662
H	-3.857100	-3.539578	1.966175
C	-3.968393	-0.639496	3.697603
H	-3.878443	-0.107340	4.641467
C	-3.340475	-1.877332	3.409673
H	-2.607436	-2.380030	4.030382

Fig. S23k

Pd	-0.045624	-2.023837	-0.040265
Pd	2.065239	-0.096933	-0.026737
Pd	-1.984622	-0.041350	3.831141
Pd	-2.108356	0.121674	-3.761205
Pd	2.012403	-2.171853	-2.112004
Pd	3.904882	-0.165827	-2.044819
Pd	-2.190490	-2.070770	1.935501
Pd	0.058731	-0.063440	1.882385
Pd	-0.067291	0.108185	-5.597980
Pd	-3.948209	0.196739	-1.856635
Pd	2.046688	-2.260386	1.977041
Pd	3.971793	-0.255083	1.930140
Pd	0.104310	-0.137504	5.597538
Pd	-2.237638	-1.999623	-1.962019
Pd	0.010901	0.009607	-1.883931
Pd	-3.878640	0.141293	1.989474
Pd	3.841372	-2.328058	-0.088084
Pd	-0.029333	-2.104529	3.756754
Pd	2.117142	-0.133092	3.797596
Pd	-0.124427	-1.929475	-3.839350
Pd	2.007666	0.035184	-3.861789
Pd	-2.004362	0.044408	0.028130
Pd	0.033621	2.096100	-3.744186

Pd	0.153276	1.939338	3.834126
Pd	-3.919712	2.314956	0.107170
Pd	2.219281	2.057090	-1.969578
Pd	-1.938661	2.139336	2.067469
Pd	2.285457	1.958558	1.990125
Pd	-2.001957	2.200982	-1.901488
Pd	0.138449	1.962774	0.034764
Pd	0.205247	3.918261	-1.846066
Pd	0.253872	3.833776	2.000949
Pd	-1.907419	4.171251	0.093558
Pd	-0.180013	-3.952870	1.897204
Pd	-0.214588	-3.859570	-2.057443
Pd	1.882455	-4.119488	-0.103364
Pd	4.185633	1.874745	-0.017354
Pd	2.418496	3.878258	0.058097
Pd	-2.351900	-3.922994	-0.051209
Pd	-4.173752	-1.979124	0.021438
Pd	-0.304602	-5.723662	-0.123595
Pd	-5.778652	0.210453	0.104149
Pd	0.419587	5.727911	0.117527
Pd	5.740787	-0.361272	-0.095759
C	-3.852079	4.262966	-0.345537
H	-4.418931	4.881591	0.368490
H	-4.072515	4.494067	-1.396464

Fig. S23l

Pd	0.964357	1.725797	0.002053
Pd	-0.106840	0.023057	-1.889568
Pd	-3.291294	2.239026	2.018973
Pd	3.119200	-2.191711	1.878750
Pd	2.791827	0.562208	-1.956916
Pd	1.605898	-1.128182	-3.764367
Pd	-0.640807	2.973019	2.029728
Pd	-1.749909	1.146933	0.000691
Pd	4.613662	-3.338860	-0.002207
Pd	1.602115	-1.135447	3.761697
Pd	-0.639016	2.976927	-2.024097
Pd	-1.735213	1.181689	-3.837922
Pd	-4.801497	3.213971	0.003060
Pd	2.790944	0.559165	1.959732
Pd	1.517034	-1.095195	-0.000543
Pd	-1.736353	1.172278	3.839418
Pd	1.039757	1.700966	-3.863759
Pd	-2.226311	4.014006	0.003244
Pd	-3.290476	2.243970	-2.015301
Pd	4.628486	-0.566953	0.001089
Pd	3.120716	-2.187981	-1.881185
Pd	-0.107907	0.018500	1.889294
Pd	1.943572	-4.084374	-0.003238
Pd	-4.469634	0.530091	0.000305
Pd	-1.170438	-1.758156	3.801620
Pd	0.438984	-2.913748	-1.912017
Pd	-2.997361	-0.549732	2.111797
Pd	-2.997981	-0.543123	-2.114357
Pd	0.436971	-2.917429	1.906058
Pd	-1.332429	-1.668328	-0.002818
Pd	-0.717138	-4.539725	-0.005242
Pd	-4.059823	-2.058415	-0.003505
Pd	-2.348478	-3.272621	1.999922
Pd	0.375432	4.530290	0.004418
Pd	3.870356	2.052803	0.003100
Pd	2.105932	3.219400	-1.956999
Pd	-1.167054	-1.747537	-3.804894
Pd	-2.346781	-3.267039	-2.008532
Pd	2.104440	3.215671	1.964518
Pd	1.037372	1.693294	3.868220
Pd	3.085614	4.768601	0.005645
Pd	-0.115785	-0.047376	5.604348
Pd	-3.435754	-4.694559	-0.006460
Pd	-0.113528	-0.033829	-5.604739
C	6.124446	-0.209432	1.404318
C	7.391237	0.155279	0.674344
C	7.391598	0.154997	-0.671027
C	6.125049	-0.209435	-1.401574
H	6.190729	-1.155530	1.962161
H	8.278013	0.411858	1.258969
H	8.278755	0.411141	-1.255271
H	6.191587	-1.154987	-1.960289
H	5.751301	0.578404	2.072789
H	5.751913	0.578974	-2.069394

Fig. S28 0 metallacycle

Pd	-0.429284	-0.033755	-1.309973
Pd	0.394295	1.942913	0.430378
Pd	4.090290	-1.623122	-1.212097
Pd	-2.677229	-1.991089	2.373622
Pd	-2.318047	1.911048	-0.473294
Pd	-1.386201	3.803959	1.296087
Pd	1.447571	-1.991785	-2.199385
Pd	2.191191	0.144466	-0.411940
Pd	-0.756735	-3.873831	1.534916
Pd	1.287303	2.052172	-2.286674
Pd	1.953195	4.001861	-0.443730
Pd	-2.034988	-2.289268	-0.394352
Pd	-1.147994	-0.066764	1.334015
Pd	2.467768	-3.704417	-0.217596
Pd	-0.599931	3.998278	-1.293568
Pd	3.121243	0.089050	-3.081442
Pd	3.865858	2.073865	-1.354341
Pd	-3.868038	-0.351277	0.384889
Pd	-3.034623	1.624248	2.221784
Pd	0.676025	-1.837125	0.533216
Pd	-1.863039	-0.038916	4.147359
Pd	4.896344	0.362381	0.491843
Pd	1.786863	-3.642650	2.495679
Pd	-0.305748	1.944245	3.181168
Pd	3.354218	-1.612556	1.531303
Pd	3.089398	2.175195	1.398851
Pd	0.028191	-1.771383	3.336190
Pd	1.552312	0.198186	2.377863
Pd	0.416160	-0.090276	-3.949606
Pd	-3.030042	-0.011491	-2.373255
Pd	-1.342767	1.960491	-3.118129
Pd	1.208036	3.971361	2.210793
Pd	-1.344793	-2.094093	-3.063865
Pd	-0.066943	-4.045470	-1.125063
C	-7.087895	2.279693	-0.254110
C	-6.267189	1.503311	-1.093450
C	-6.819488	0.375781	-1.726082
C	-8.167243	0.023756	-1.557127
C	-8.980034	0.813662	-0.720693
C	-8.441461	1.937802	-0.064120
H	-6.671021	3.154498	0.238647
H	-5.229037	1.774683	-1.269827
H	-8.563274	-0.840828	-2.082046
H	-10.026257	0.551564	-0.585285
H	-9.070316	2.544856	0.581674
O	-6.050566	-0.391794	-2.645581
C	-4.976505	-1.195785	-2.203172
C	-4.191233	-1.766340	-3.260009
C	-4.979307	-1.757681	-0.849398
C	-3.367619	-2.940854	-3.020695
H	-4.435275	-1.480794	-4.279321
C	-4.217640	-2.986915	-0.654243
H	-5.895975	-1.641512	-0.264297
C	-3.429026	-3.562923	-1.700828
H	-3.151778	-3.586498	-3.877723
H	-4.441330	-3.582542	0.226199
H	-3.076906	-4.584965	-1.595416

Fig. S28 1 metallacycle

Pd	0.594861	-0.234814	-1.126136
Pd	-0.318714	-1.664525	1.103532
Pd	-4.032962	0.546623	-2.038215
Pd	1.937350	3.068904	1.954296
Pd	2.471170	-1.492523	0.469136
Pd	1.541664	-2.849445	2.676147
Pd	-1.313356	1.014759	-2.807066
Pd	-2.113993	-0.534865	-0.494247
Pd	-0.020512	4.296799	0.347773
Pd	-0.677311	-2.733649	-1.536777
Pd	-1.458680	-4.118153	0.755007
Pd	1.707334	2.422735	-0.852590
Pd	0.810929	0.732036	1.433784
Pd	-2.895675	3.077368	-1.629850
Pd	1.169303	-3.889849	0.149550
Pd	-2.530441	-1.453780	-3.122328
Pd	-3.406401	-2.884575	-0.923140
Pd	3.591068	1.098814	0.688277
Pd	2.696796	-0.241553	3.046632
Pd	-1.032903	1.856096	-0.108842
Pd	1.014245	1.694564	4.175524

Pd	-4.905486	-0.879637	0.111265
Pd	-2.645295	3.974633	0.998786
Pd	-0.095556	-0.717999	3.739775
Pd	-3.802094	1.535255	0.613429
Pd	-3.070212	-2.036588	1.761942
Pd	-0.871232	2.778616	2.616420
Pd	-1.975272	0.394664	2.198748
Pd	0.241724	-1.150674	-3.692572
Pd	3.309595	-0.208509	-1.889804
Pd	2.107177	-2.652355	-2.225132
Pd	-1.117936	-3.188554	3.333235
Pd	1.505967	1.350524	-3.367107
Pd	-0.298441	3.511139	-2.310240
C	7.213460	-0.960543	1.342106
C	6.422432	-0.609361	0.232601
C	6.957186	0.261033	-0.733449
C	8.262787	0.764241	-0.625523
C	9.047517	0.397912	0.485033
C	8.523656	-0.459437	1.472709
H	6.808420	-1.633111	2.094155
H	5.420308	-1.014571	0.108619
H	8.647914	1.417006	-1.403454
H	10.060837	0.780665	0.575998
H	9.130611	-0.739484	2.329514
O	6.235875	0.585700	-1.915184
C	5.017325	1.298504	-1.866757
C	4.356020	1.416734	-3.134531
C	4.744155	2.222840	-0.761145
C	3.381191	2.470012	-3.363804
H	4.794936	0.897871	-3.981783
C	3.836077	3.328864	-1.044658
H	5.564740	2.419506	-0.065394
C	3.163150	3.449148	-2.300744
H	3.236848	2.803208	-4.396179
H	3.852348	4.179655	-0.369610
H	2.688201	4.391426	-2.557948
C	2.479354	-3.084104	-4.228867
C	3.647824	-4.009816	-1.847957
C	4.072003	-4.625896	-3.158423
C	3.523445	-4.172099	-4.300093
H	3.286143	-4.754932	-1.118011
H	4.823206	-5.420401	-3.150223
H	1.503294	-3.402146	-4.635516
H	4.439102	-3.410961	-1.374603
H	2.795983	-2.150368	-4.716398
H	3.805912	-4.565338	-5.280365

Fig. S28 3 metallacycle

C	-4.318888	-2.768520	-2.388657
C	-5.016717	-0.230198	-3.359948
C	-5.861856	-1.416888	-3.751340
C	-5.528442	-2.634644	-3.283026
H	-4.644593	0.340621	-4.228342
H	-6.726097	-1.261014	-4.402911
H	-3.620592	-3.547241	-2.762610
H	-5.534661	0.458285	-2.677638
H	-4.569690	-3.020449	-1.348413
H	-6.101320	-3.531419	-3.535533
Pd	-0.034330	-0.469458	1.280845
Pd	0.566235	-1.567374	-1.169822
Pd	4.445516	1.123941	1.215848
Pd	-2.871580	3.098813	-1.102046
Pd	-2.018273	-1.925574	-0.000335
Pd	-1.378461	-2.973976	-2.530019
Pd	2.001525	1.069104	2.640264
Pd	2.454526	-0.181580	0.067906
Pd	-0.627225	4.179162	0.260947
Pd	1.718965	-2.766248	1.135936
Pd	2.155612	-3.767908	-1.396934
Pd	-1.744748	1.922177	1.385746
Pd	-1.162538	0.553208	-0.974712
Pd	2.790697	3.386732	1.339510
Pd	-0.317866	-4.108546	-0.246075
Pd	3.696377	-1.191769	2.442632
Pd	4.144664	-2.231432	-0.037293
Pd	-3.742229	0.346335	0.264540
Pd	-3.346805	-0.912859	-2.330199
Pd	0.877805	1.922204	0.181425
Pd	-2.357480	1.501122	-3.241046
Pd	5.008196	0.083756	-1.272759
Pd	1.751871	4.364055	-1.046472

Pd	-0.564255	-0.542316	-3.562805
Pd	3.382659	2.227682	-1.212042
Pd	3.058676	-1.311958	-2.515402
Pd	-0.207836	2.910227	-2.249620
Pd	1.456635	0.820362	-2.423208
Pd	1.080493	-1.397013	3.547065
Pd	-2.432726	-0.863823	2.550743
Pd	-0.806997	-3.152983	2.360242
Pd	1.076295	-2.708269	-3.715382
Pd	-0.605393	0.857087	3.760310
Pd	0.389675	3.320567	2.651407
C	-7.172499	-2.008510	0.510107
C	-6.090478	-1.673743	1.345762
C	-6.353219	-1.085094	2.594719
C	-7.666052	-0.856163	3.035782
C	-8.740892	-1.209987	2.197954
C	-8.496533	-1.778770	0.932173
H	-6.979272	-2.447525	-0.465158
H	-5.065287	-1.873704	1.042933
H	-7.828753	-0.418633	4.016386
H	-9.761019	-1.039130	2.532323
H	-9.327200	-2.044513	0.283867
O	-5.309754	-0.782533	3.511194
C	-4.298841	0.161087	3.216611
C	-3.366516	0.361815	4.292800
C	-4.494911	1.171118	2.164078
C	-2.582159	1.576020	4.373333
H	-3.449542	-0.287972	5.158339
C	-3.760376	2.421769	2.325354
H	-5.496505	1.240396	1.727088
C	-2.825170	2.619690	3.386798
H	-2.212011	1.877138	5.357652
H	-4.107393	3.286424	1.768285
H	-2.466050	3.623414	3.592479
C	-2.830026	4.837317	-2.246567
C	-4.931328	3.349019	-1.207673
C	-5.259863	4.579151	-2.017472
C	-4.240966	5.283258	-2.541697
H	-5.295206	3.405596	-0.169803
H	-6.305971	4.859695	-2.165261
H	-2.283212	5.547857	-1.602537
H	-5.298878	2.419722	-1.666490
H	-2.231868	4.657713	-3.152617
H	-4.396651	6.169726	-3.162038
C	-0.257580	-4.198016	4.067846
C	-2.192436	-4.709199	2.107165
C	-1.971443	-5.727538	3.196572
C	-1.077333	-5.459546	4.166322
H	-2.166689	-5.142775	1.092819
H	-2.560384	-6.648578	3.185784
H	0.814181	-4.395467	3.903146
H	-3.136316	-4.153643	2.222209
H	-0.383557	-3.522683	4.928098
H	-0.913656	-6.130889	5.013512

Fig. S28 4 metallacycle

C	-4.910867	-2.650673	-1.747623
C	-5.187274	-0.222783	-3.134490
C	-6.181346	-1.328839	-3.390463
C	-6.050870	-2.492617	-2.724203
H	-4.740980	0.173702	-4.065192
H	-6.988762	-1.159386	-4.108096
H	-4.338769	-3.585350	-1.926190
H	-5.615345	0.614308	-2.565993
H	-5.225669	-2.645730	-0.694619
H	-6.741138	-3.327637	-2.872570
Pd	-0.007588	-0.254742	1.345508
Pd	0.171298	-1.971301	-0.827050
Pd	4.594050	0.592135	0.673421
Pd	-2.561668	3.173697	-1.604546
Pd	-2.324488	-1.629927	0.471125
Pd	-2.024138	-3.318488	-1.733550
Pd	2.317525	1.190375	2.198343
Pd	2.339868	-0.666885	-0.051532
Pd	-0.031369	4.048551	-0.678781
Pd	1.320354	-2.784869	1.646546
Pd	1.395635	-4.379361	-0.641200
Pd	-1.399210	2.332509	0.998040
Pd	-1.174160	0.417829	-0.986651
Pd	3.446841	3.148416	0.321135
Pd	-0.981262	-4.066483	0.718331

Pd	3.570245	-1.252921	2.392465
Pd	3.685154	-2.967922	0.251272
Pd	-3.713720	0.882809	0.243030
Pd	-3.654453	-0.997289	-1.956692
Pd	1.160232	1.695176	-0.302413
Pd	-2.426933	1.047070	-3.329387
Pd	4.816537	-1.116972	-1.435493
Pd	2.190047	3.508853	-2.266268
Pd	-0.979766	-1.273395	-3.309453
Pd	3.544586	1.227806	-1.873687
Pd	2.575245	-2.389654	-2.316046
Pd	-0.065642	2.227267	-2.858915
Pd	1.294316	-0.047086	-2.602672
Pd	1.074559	-0.866706	3.707451
Pd	-2.371198	0.046829	2.651290
Pd	-1.165555	-2.467088	3.022033
Pd	0.329345	-3.653092	-3.112381
Pd	-0.230370	1.640893	3.487820
Pd	1.075112	3.622924	1.936692
C	-7.437088	-0.742490	0.979901
C	-6.282853	-0.471564	1.738633
C	-6.368886	0.446287	2.799050
C	-7.578761	1.074234	3.132328
C	-8.730207	0.784914	2.374493
C	-8.659682	-0.117225	1.294554
H	-7.380020	-1.439212	0.147660
H	-5.338791	-0.964738	1.522180
H	-7.609083	1.761743	3.972679
H	-9.673601	1.262455	2.626780
H	-9.547694	-0.334820	0.707065
O	-5.247867	0.703056	3.636606
C	-4.131586	1.429308	3.171256
C	-3.105110	1.622747	4.155832
C	-4.197671	2.238002	1.957432
C	-2.128361	2.683127	4.004365
H	-3.237007	1.143589	5.121138
C	-3.240071	3.335971	1.855767
H	-5.182295	2.391563	1.505733
C	-2.242465	3.563617	2.856399
H	-1.655378	3.071788	4.911369
H	-3.461952	4.130415	1.149510
H	-1.710290	4.509528	2.865295
C	3.440433	5.281389	0.400918
C	2.543045	4.375387	3.298248
C	3.205726	5.578393	2.898244
C	3.859569	5.794992	1.668933
H	3.114588	3.447957	3.115054
H	3.284065	6.374786	3.644900
H	4.036864	5.583028	-0.465901
H	2.065636	4.391741	4.281819
H	2.357012	5.309075	0.216301
H	4.700894	6.493920	1.669583
C	-2.368413	4.596677	-3.107298
C	-4.582978	3.651344	-1.735002
C	-4.799035	4.699962	-2.799671
C	-3.725428	5.144734	-3.476653
H	-4.880217	3.995543	-0.731060
H	-5.810555	5.065686	-2.995854
H	-1.706422	5.357609	-2.659049
H	-5.098865	2.704430	-1.951604
H	-1.840859	4.135451	-3.956819
H	-3.799029	5.889936	-4.273346
C	-0.764537	-3.005182	4.985325
C	-2.744914	-3.837761	3.182125
C	-2.642456	-4.522588	4.521672
C	-1.719456	-4.095568	5.403424
H	-2.778001	-4.545961	2.335119
H	-3.335410	-5.335969	4.753430
H	0.284265	-3.345186	4.955304
H	-3.615916	-3.168925	3.105722
H	-0.842897	-2.095723	5.600345
H	-1.630014	-4.518896	6.407541

Supplementary References

1. Gurrath, M. *et al.* Palladium catalysts on activated carbon supports. *Carbon* **38**, 1241-1255 (2000).
2. Scholten, J. J. F., Pijpers, A. P. & Hustings, A. M. L. Surface Characterization of Supported and Nonsupported Hydrogenation Catalysts. *Catalysis Reviews* **27**, 151-206 (1985).
3. Ichikawa, S., Hoppa, H. & Boudart, M. Disproportionation of CO on small particles of silica-supported palladium. *J. Catal.* **91**, 1-10 (1985).
4. Peng, J. & Wang, S. Performance and characterization of supported metal catalysts for complete oxidation of formaldehyde at low temperatures. *Appl. Catal. B-Environ.* **73**, 282-291 (2007).
5. Guo, L. *et al.* Theoretical insight into an empirical rule about organic corrosion inhibitors containing nitrogen, oxygen, and sulfur atoms. *Appl. Surf. Sci.* **406**, 301-306 (2017).
6. Grimblat, N. & Sarotti, A. M. Computational Chemistry to the Rescue: Modern Toolboxes for the Assignment of Complex Molecules by GIAO NMR Calculations. *Chemistry* **22**, 12246-12261 (2016).
7. Omar, S. *et al.* Density Functional Theory Analysis of Dichloromethane and Hydrogen Interaction with Pd Clusters: First Step to Simulate Catalytic Hydrodechlorination. *J. Phys. Chem. C* **115**, 14180-14192 (2011).
8. Fortea-Perez, F. R. *et al.* The MOF-driven synthesis of supported palladium clusters with catalytic activity for carbene-mediated chemistry. *Nat. Mater.* **16**, 760-766 (2017).
9. Szeleszczuk, Ł., Pisklak, D. M., Zielińska-Pisklak, M. & Wawer, I. Effects of structural differences on the NMR chemical shifts in cinnamic acid derivatives: Comparison of GIAO and GIPAW calculations. *Chemical Physics Letters* **653**, 35-41 (2016).
10. Wu, D. *et al.* Lignin Compounds to Monoaromatics: Selective Cleavage of C-O Bonds over a Brominated Ruthenium Catalyst. *Angew. Chem. Int. Ed.* **60**, 12513-12523 (2021).
11. Chatterjee, M., Chatterjee, A., Ishizaka, T. & Kawanami, H. Rhodium-mediated hydrogenolysis/hydrolysis of the aryl ether bond in supercritical carbon dioxide/water: an experimental and theoretical approach. *Catal. Sci. Technol.* **5**, 1532-1539 (2015).
12. Guo, M., Peng, J., Yang, Q. & Li, C. Highly Active and Selective RuPd Bimetallic NPs for the Cleavage of the Diphenyl Ether C–O Bond. *ACS Catal.* **8**, 11174-11183 (2018).
13. Hong, Y. *et al.* Synergistic Catalysis between Pd and Fe in Gas Phase Hydrodeoxygenation of m-Cresol. *ACS Catal.* **4**, 3335-3345 (2014).
14. Ferrari, A. C. Raman spectroscopy of graphene and graphite: Disorder, electron–phonon coupling, doping and nonadiabatic effects. *Solid State Communications* **143**, 47-57 (2007).
15. Kang, D. *et al.* Oxidation resistance of iron and copper foils coated with reduced graphene oxide multilayers. *ACS Nano* **6**, 7763-7769 (2012).
16. Shebanova, O. N. & Lazor, P. Raman study of magnetite (Fe3O4): laser-induced thermal effects and oxidation. *J. Raman Spectrosc.* **34**, 845-852 (2003).
17. Ferrari, A. C. & Robertson, J. Resonant Raman spectroscopy of disordered, amorphous, and diamondlike carbon. *Phys. Rev. B* **64** (2001).
18. de Smit, E. *et al.* Stability and reactivity of -chi-theta iron carbide catalyst phases in Fischer-Tropsch synthesis: controlling mu(C). *J. Am. Chem. Soc.* **132**, 14928-14941 (2010).
19. Hensen, E., Zhu, Q., Liu, P., Chao, K. & Vansanten, R. On the role of aluminum in the selective oxidation of benzene to phenol by nitrous oxide over iron-containing MFI zeolites: an in situ Fe XANES study. *J. Catal.* **226**, 466-470 (2004).
20. Nassiri, H. *et al.* Water shifts PdO-catalyzed lean methane combustion to Pt-catalyzed rich combustion in Pd–Pt catalysts: In situ X-ray absorption spectroscopy. *J. Catal.* **352**, 649-656 (2017).

21. Li, Z. *et al.* Mono-disperse PdO nanoparticles prepared via microwave-assisted thermo-hydrolyzation with unexpectedly high activity for formic acid oxidation. *Electrochimica Acta* **329** (2020).
22. Wu, C. T. *et al.* A non-syn-gas catalytic route to methanol production. *Nat. Commun.* **3**, 1050 (2012).
23. Murayama, M., Howe, J. M., Hidaka, H. & Takaki, S. Atomic-level observation of disclination dipoles in mechanically milled, nanocrystalline Fe. *Science* **295**, 2433-2435 (2002).
24. Chang, Q. *et al.* Relationship between Iron Carbide Phases (ε-Fe2C, Fe7C3, and χ-Fe5C2) and Catalytic Performances of Fe/SiO2 Fischer–Tropsch Catalysts. *ACS Catal.* **8**, 3304-3316 (2018).
25. de Smit, E. & Weckhuysen, B. M. The renaissance of iron-based Fischer-Tropsch synthesis: on the multifaceted catalyst deactivation behaviour. *Chem Soc Rev* **37**, 2758-2781 (2008).
26. Ziemecki, S. B., Jones, G. A., Swartzfager, D. G., Harlow, R. L. & Faber, J. Formation of interstitial palladium-carbon phase by interaction of ethylene, acetylene, and carbon monoxide with palladium. *J. Am. Chem. Soc.* **107**, 4547-4548 (1985).
27. Krüger, S., Vent, S., Nörtemann, F., Staufer, M. & Rösch, N. The average bond length in Pd clusters Pdn, n=4–309: A density-functional case study on the scaling of cluster properties. *The Journal of Chemical Physics* **115**, 2082-2087 (2001).
28. Neyman, K. M. & Schauermann, S. Hydrogen diffusion into palladium nanoparticles: pivotal promotion by carbon. *Angew. Chem. Int. Ed.* **49**, 4743-4746 (2010).
29. Rodriguez, N. M. *et al.* XPS, EM, and Catalytic Studies of the Accumulation of Carbon on Pt Black. *J. Catal.* **197**, 365-377 (2001).
30. Wild, U., Pfänder, N. & Schlögl, R. Species analysis of automotive carbon particles: Application of XPS for integral analysis of filter samples. *Fresenius Journal of Analytical Chemistry* **357**, 420-428 (1997).
31. Opitz, A. K. *et al.* Enhancing electrochemical water-splitting kinetics by polarization-driven formation of near-surface iron(0): an in situ XPS study on perovskite-type electrodes. *Angew. Chem. Int. Ed.* **54**, 2628-2632 (2015).
32. Zhang, J. *et al.* Surface engineering of earth-abundant Fe catalysts for selective hydrodeoxygenation of phenolics in liquid phase. *Chem. Sci.* **11**, 5874-5880 (2020).
33. Ivanciuc, O., Rabine, J. P., Cabrol-Bass, D., Panaye, A. & Doucet, J. P. 13C NMR Chemical Shift Prediction of sp2 Carbon Atoms in Acyclic Alkenes Using Neural Networks. *Journal of Chemical Information and Computer Sciences* **36**, 644-653 (1996).
34. Ye, J. *et al.* Remote C-H alkylation and C-C bond cleavage enabled by an in situ generated palladacycle. *Nat. Chem.* **9**, 361-368 (2017).
35. Zhan, B. B. *et al.* Site-Selective delta-C(sp(3))-H Alkylation of Amino Acids and Peptides with Maleimides via a Six-Membered Palladacycle. *Angew. Chem. Int. Ed.* **57**, 5858-5862 (2018).
36. Ormaza, M. *et al.* Controlled spin switching in a metallocene molecular junction. *Nat. Commun.* **8**, 1974 (2017).
37. Yamamoto, K., Kimura, S. & Murahashi, T. sigma-pi Continuum in Indole-Palladium(II) Complexes. *Angew. Chem. Int. Ed.* **55**, 5322-5326 (2016).
38. Bunting, P., Chisholm, M. H., Gallucci, J. C. & Lear, B. J. Extent of M2 delta to ligand pi-conjugation in neutral and mixed valence states of bis(4-isonicotinate)-bis(2,4,6-triisopropylbenzoate) dimetal complexes (MM), where M = Mo or W, and their adducts with tris(pentafluorophenyl)boron. *J. Am. Chem. Soc.* **133**, 5873-5881 (2011).
39. Sprengers, J. W., Wassenaar, J., Clement, N. D., Cavell, K. J. & Elsevier, C. J. Palladium-(N-Heterocyclic Carbene) Hydrogenation Catalysts. *Angew. Chem. Int. Ed.* **117**, 2062-2065 (2005).

40. Wilson, E. B. The Normal Modes and Frequencies of Vibration of the Regular Plane Hexagon Model of the Benzene Molecule. *Phys. Rev.* **45**, 706-714 (1934).
41. Busca, G., Zerlia, T., Lorenzelli, V. & Girelli, A. Fourier transform-infrared study of the adsorption of unsaturated and aromatic hydrocarbons on the surface of α-Fe2O3. Part III. Toluene, ethylbenzene and styrene. *React. Kinet. Catal. Lett.* **27**, 429-432 (1985).
42. Painter, P. C. & Koenig, J. L. A normal vibrational analysis of benzene. *Spectrochimica Acta Part A: Molecular Spectroscopy* **33**, 1019-1024 (1977).
43. Gabashvili, I. S., Menikh, A., Ségui, J. & Fragata, M. Protein structure of photosystem II studied by FT-IR spectroscopy. Effect of digalactosyldiacylglycerol on the tyrosine side chain residues. *J. Mol. Struct.* **444**, 123-133 (1998).
